# Cellular and molecular basis of thyroid autoimmunity

**DOI:** 10.1530/ETJ-21-0024

**Published:** 2021-11-01

**Authors:** Joanna Bogusławska, Marlena Godlewska, Ewa Gajda, Agnieszka Piekiełko-Witkowska

**Affiliations:** 1 Centre of Postgraduate Medical Education, Department of Biochemistry and Molecular Biology, Warsaw, Poland

**Keywords:** thyroid autoimmunity, autoimmune thyroid disease, AITD, Hashimoto’s thyroiditis, Graves’ disease, thyroid antigens, non-coding RNAs, miRNAs, circRNAs, lncRNAs, microbiome

## Abstract

Autoimmune thyroid disease (AITD) is the most common human autoimmune disease. The two major clinical manifestations of AITD are Graves’ disease and Hashimoto’s thyroiditis (HT). AITD is characterized by lymphocytic infiltration of the thyroid gland, leading either to follicular cell damage, thyroid gland destruction, and development of hypothyroidism (in HT) or thyroid hyperplasia, induced by thyroid antibodies which activate thyrotropin receptor (TSHR) on thyrocytes, leading to hyperthyroidism. The aim of this review is to present up-to-date picture of the molecular and cellular mechanisms that underlie the pathology of AITD. Based on studies involving patients, animal AITD models, and thyroid cell lines, we discuss the key events leading to the loss of immune tolerance to thyroid autoantigens as well as the signaling cascades leading to the destruction of thyroid gland. Special focus is given on the interplay between the environmental and genetic factors, as well as ncRNAs and microbiome contributing to AITD development. In particular, we describe mechanistic models by which SNPs in genes involved in immune regulation and thyroid function, such as CD40, TSHR, FLT3, and PTPN22, underlie AITD predisposition. The clinical significance of novel diagnostic and prognostic biomarkers based on ncRNAs and microbiome composition is also underscored. Finally, we discuss the possible significance of probiotic supplementation on thyroid function in AITD.

## Introduction

Autoimmune thyroid disease (AITD) is defined as a dysregulation of the immune system leading to autoimmune attack on the thyroid gland. It is the most common autoimmune disease affecting humans (1). The two major clinical manifestations of AITD are Graves’ disease (GD) and Hashimoto’s thyroiditis (HT). The additional, less prevalent AITDs include postpartum thyroiditis, drug-induced thyroiditis, thyroiditis associated with polyglandular autoimmune syndromes (2). Both HT and GD are characterized by lymphocytic infiltration of the thyroid gland, which, however, differently affects thyroid function. In the case of HT, the resulting inflammation leads to follicular cell damage, thyroid gland destruction, and development of hypothyroidism. In contrast, GD is mainly associated with hyperthyroidism, resulting from the presence of thyroid-stimulating antibodies which activate thyrotropin receptor (TSHR) on thyrocytes, leading to thyroid hyperplasia. Only a minor number of GD patients develop hypothyroidism due to the TSHR-blocking antibodies (1, 2). Of note, hypothyroidism in GD patients can be observed as a short-term effect of the blocking autoantibodies, as well as a longer effect due to eventual autoimmune thyroid destruction (3).

The knowledge of the molecular AITD background comes from patient observations, animal AITD models, as well as *in vitro* experiments performed on thyroid cell lines. The mouse AITD models are achieved through classical immunization of susceptible mice with the thyroid autoantigens or by more advanced techniques such as use of adenovirus vector carrying the human sequences of key thyroid autoantigens. The topic of mouse AITD models is very broad and due to manuscript text limitations, we cannot discuss it here in detail. The reader can further explore this subject in the previously published excellent review articles (1, 4).

The aim of this review is to present the up-to-date picture of the molecular and cellular mechanisms that underlie the pathology of AITD. Our intention is to inspire basic researchers to initiate more in-depth studies on the molecular biology of AITD. We also hope that our article will complement the clinical view of HT and GD, recently presented in several outstanding papers (5, 6, 7, 8).

## Thyroid antigens in AITD

The autoantigens targeted during autoimmune attack in AITD are proteins expressed by the thyroid tissue, indispensable for its physiological function. The key features of thyroid autoantigens are presented below.

### Thyrotropin receptor (TSHR)

**Figure 1:**
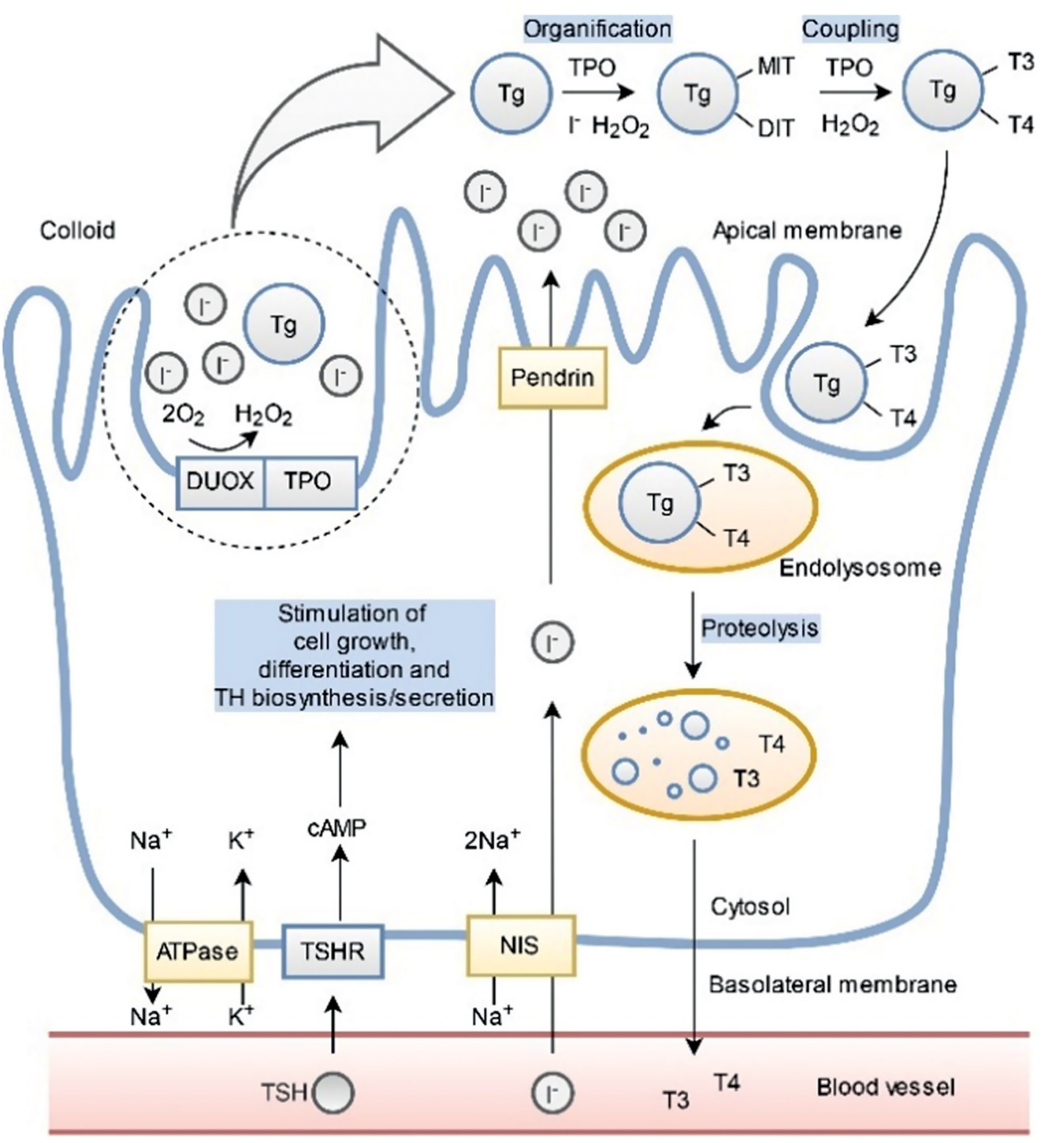
Localization and physiological function of thyroid antigens in thyrocytes. The sodium iodide symporter (NIS) transports I^−^ and Na^+^ through the basolateral plasma membrane of a thyroid epithelial cell. The Na^+^/K^+^ ATPase pump maintains the sodium diffusion gradient required for NIS function. Pendrin participates in the apical iodide efflux into the colloid of thyroid follicle. Thyroid peroxidase (TPO) catalyzes iodination of tyrosines in thyroglobulin (Tg), which attaches one or two iodine to form monoiodotyrosine (MIT) or diiodotyrosine (DIT), respectively. TPO also catalyzes the coupling of iodotyrosine residues to form triiodothyronine (T3) and thyroxine (T4) attached to Tg, whereas the dual oxidase (DUOX) supplies hydrogen peroxide (H_2_O_2_) for thyroid hormones (TH) biosynthesis. Release of TH requires engulfing colloid material (endocytosis) to form intracellular endosomes (not shown) that merge with lysosomes to form an endolysosome. TH liberated from the Tg scaffold are subsequently secreted into the blood vessels. Binding of thyrotropin (TSH; or thyroid-stimulating antibody) to TSH receptor (TSHR) activates intracellular signaling by the cAMP pathway leading to thyrocytes growth, differentiation, as well as production and release of TH.

TSHR is indispensable for thyrotropin (TSH) signal transduction, thyroid hormones (TH) production, and proliferation of follicular epithelial cells. TSHR, primarily expressed on the basolateral membrane of thyrocytes (Fig. 1), belongs to the G protein-coupled receptors. TSHR consists of the extracellular leucine-rich repeat domain (LRD) that is linked by a hinge region (HinR) to the transmembrane-spanning domain. The single polypeptide chain TSHR precursor undergoes intramolecular cleavage within the HinR. Due to the post-translational processing, the TSHR comprises an extracellular, heavily glycosylated A-subunit and a transmembrane and intracellular B-subunit coupled by disulphide bridges. Autoantibodies to TSHR are directly involved in the pathophysiology of AITD and their measurement is recommended for early diagnosis and management of patients with GD (9). Classical biochemical features of hyperthyroid GD, including elevated TH levels and undetectable TSH, arise from the action of TSHR-stimulating antibodies (TSAb), which act as TSHR agonists by stimulating thyroid growth and TH production in an unregulated manner. Contrary, TSHR-blocking antibodies (TBAb) act as antagonists, which block the action of the TSH, leading to the HT hypothyroidism. Finally, neutral antibodies to TSHR, which are also detected in GD sera, bind to the receptor without any influence on its activity (10). The complexity of the TSHR’s tertiary and quaternary structure contributes to the difficulty in delineating the epitopes recognized by TSHR-specific autoantibodies. It seems that all TSAb and most TBAb bind to conformational epitopes formed by residues that are sequentially discontinuous but close together in 3D space, whereas neutral antibodies to TSHR recognize linear epitopes localized predominantly within HinR. The crystal structure of TSHR LRD bound to blocking (M22) or stimulating (K1-70) antibodies revealed that the exact antigenic sites of TSHR-specific TBAb and TSAb are strongly overlapping and directed almost exclusively to A-subunit (11, 12).

### Thyroid peroxidase (TPO)

TPO is a glycosylated heme-containing homodimer of two 107-kDa transmembrane subunits located in the apical membrane of thyrocytes facing the follicular lumen (Fig. 1). It catalyzes iodination of tyrosyl residues in thyroglobulin (Tg) and further coupling of iodotyrosine residues to form iodothyronines attached to Tg (13). TPO is made up of a large N-terminal extracellular ectodomain followed by short transmembrane and cytoplasmic regions. Ectodomain exposed to the lumen is composed of three domains: a heme-containing and catalytically active myeloperoxidase (MPO)-like domain, a complement control protein (CCP)-like domain, and an EGF-like domain (14). TPO is also a major thyroid antigen, to which both humoral and cellular immune responses are directed. TPO autoantibodies (TPOAb) occur in almost all HT patients and approximately 75% of individuals with GD, whereas their positivity in euthyroid patients may be the risk factor for future thyroid disorders (15). They may be involved in autoimmune thyroid cell death via antibody-dependent cytotoxicity cells (ADCC) and C3 complement-mediated cytotoxicity (16). Moreover, TPOAb also influence the diversity of the pathogenic T-cell epitope repertoire. The majority of TPOAb recognize overlapping conformation-dependent epitopes, called immunodominant region A (IDR-A) and B (IDR-B), located in the MPO-like domain and to a lesser extent in the CCP-like domain (17, 18). Specific patterns of TPOAb recognition are stable in an individual over time and genetically inherited in families (16, 19). Recently reported data shed new light on the interaction between TPO and autoantibodies (14, 20). Unexpectedly, it seems that monomeric TPO is preferentially recognized by autoantibodies, whereas in dimeric mature, TPO the binding sites are hidden. Additionally, the antigen–antibody interaction is connected with conformational changes in TPO that bring together previously far apart residues into a continuous epitope. This may explain the previously contradictory epitope mapping data reported by various research teams that IDR-A and IDR-B include residues theoretically too far apart to be involved by single autoantibody complementarity-determining regions based on previous modeling (14, 20).

### Thyroglobulin (Tg)

Tg is the largest and most abundant autoantigen in the thyroid gland. It is a soluble glycoprotein homodimer composed of two subunits of ~330 kDa each. Tg is the matrix for TH synthesis and is the form in which hormones are stored in the gland (Fig. 1). After endocytosis from the colloid to cytoplasmic lysosomes for subsequent proteolysis, free TH (triiodothyronine (T3) and thyroxine (T4)) are released from the Tg molecule (21). The N-terminal part of Tg contains a large number of cysteine-rich domains, which are spaced by linker domains and connected to a C-terminal domain of high homology to choline-esterase (21). Recent study (22) revealed the first atomic structure of full-length Tg and identified its hormone-forming tyrosine residues. Interestingly, only 4 out of 67 tyrosine residues in each monomer are hormonogenic (22). Anti-Tg antibodies (TgAb) may be present at high concentrations in AITD, nevertheless, they are also found in some clinically euthyroid individuals. Therefore, they are less predictive of overt thyroid dysfunction than TPOAb. A pathogenic role for TgAb has been postulated via ADCC but not through complement fixation (16). In AITD, the prevalent TgAb species recognize native (rather than denatured) antigen. TgAb bind to a number of overlapping epitopic domains located mainly in the central region and C-terminal end of Tg and no intramolecular epitope spreading is observed (similar to TPOAb). Whereas, TgAb in sera from healthy subjects do have a different epitopic pattern (23).

### Other thyroid autoantigens

The immune response to iodide transporters, sodium iodide symporter (NIS) and pendrin, has been widely discussed (24, 25). Taking into account all the available data, including the recent study of Eleftheriadou and co-workers (25), it seems that only NIS antibodies (NISAb) may play a relevant role in AITD. NISAb positivity is increased in AITD, especially in GD patients, whereas their presence in healthy donors is rare (25). NIS is a ~90-kDa glycoprotein that mediates the active transport of iodide along the basolateral membrane of thyrocyte (Fig. 1). The protein is composed of 13 transmembrane domains with the extracellular N-terminus and the C-terminal cytosolic tail. Epitopes for NISAb are likely located in the extramembranous regions of the protein and at least partly overlap. Compared to NISAb, the diagnostic value of antibodies directed against pendrin, which is an apical membrane-bound iodide transporter, seems to be low (25). The characteristics of thyroid antigens are summarized in Table 1.

**Table 1:** Characteristics of thyroid antigens.

	Tg	TSHR	TPO	NIS
Molecular weight and oligomeric state	Homodimer of ~330 kDa monomers	Dimer/oligomer of ~90 kDa monomers	Homodimer of ~107 kDa monomers	Dimer/oligomer of ~90 kDa monomers
Localization in thyrocyte	Follicular lumen	Basolateral membrane	Apical membrane	Basolateral membrane
Function	Matrix for the sequestration of iodine and synthesis of TH	Major stimulator of thyroid cell growth, differentiation and function	Iodination and coupling of the hormonogenic tyrosines in Tg	Active transport of iodide
Post-translational modifications	Iodination, phosphorylation, glycosylation, carboxymethylation, sulfation, dimerization	Intramolecular cleavage, palmitoylation, dimerization/oligomerization	N-terminal trimming, heme incorporation, glycosylation, dimerization	Glycosylation, dimerization/oligomerization
Epitope localization	Predominantly central region and C-terminus	Predominantly A-subunit	Predominantly MPO-like domain and to lesser extent CCP-like domain	Predominantly extramembranous regions

CCP, complement control protein; GD, Graves’ disease; HT, Hashimoto’s thyroiditis; MPO, myeloperoxidase; NIS, sodium iodide symporter; Tg, thyroglobulin; TH, thyroid hormones; TPO, thyroid peroxidase; TSHR, thyrotropin receptor.

## The molecular and cellular mechanisms involved in AITD

### Loss of immune tolerance

**Figure 2:**
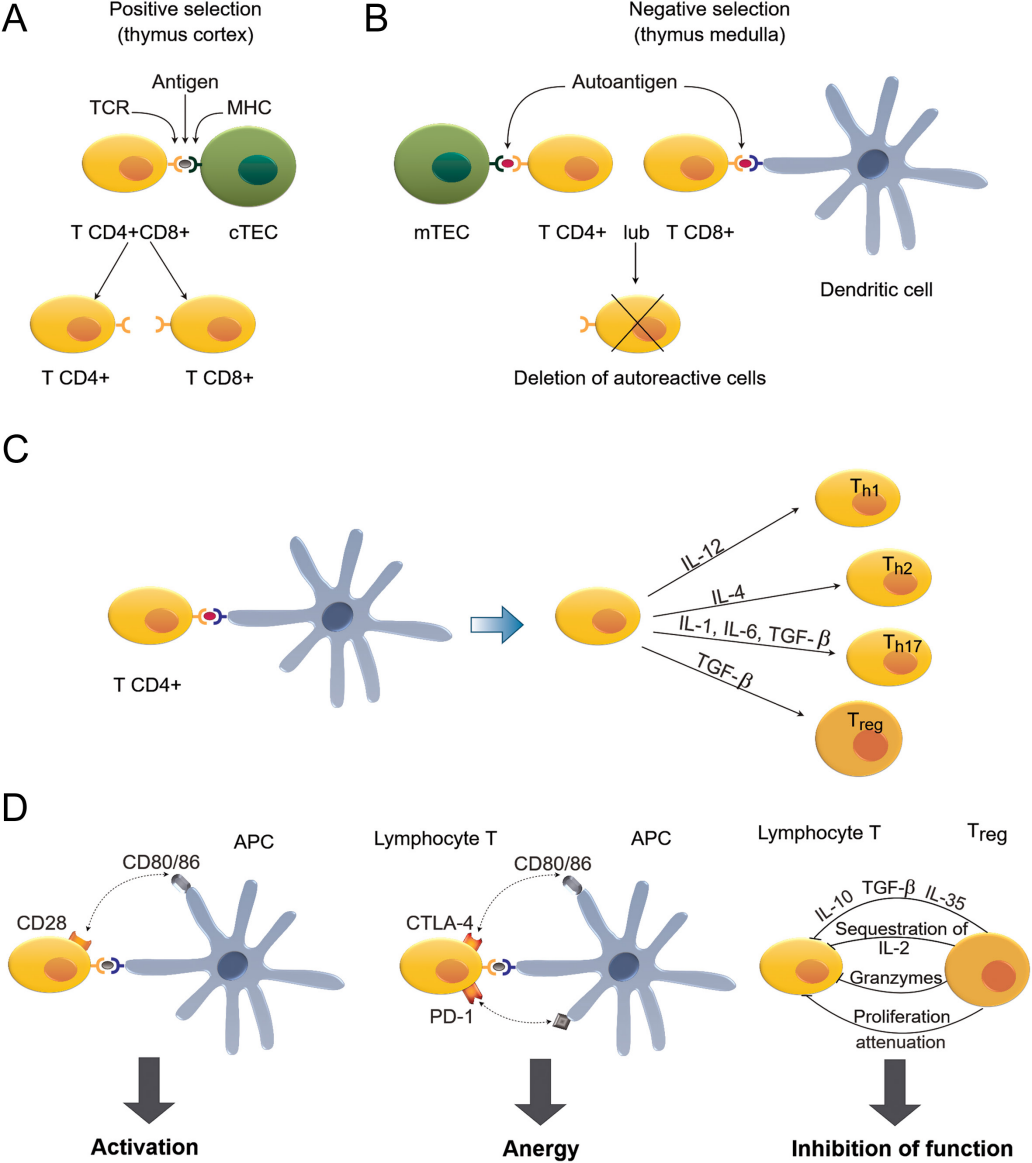
The mechanisms of selection, differentiation, and activation of T lymphocytes. (A) The positive selection of T cells (called thymocytes) expressing both CD4 and CD8 molecules in thymus cortex. Random recombination of genes encoding T cell receptor (TCR) peptides in T cell precursors leads to the expression of multiple TCR variants. TCR receptors interact with antigens presented by cortical thymic epithelial cells (cTECs) in complex with major histocompatibility complex (MHC) molecules. This interaction triggers the process of lymphocyte differentiation, leading to the generation of T cells expressing either CD4 or CD8 glycoprotein receptors on their surface. (B) The unwanted effect of TCR genes’ recombination is the production of receptor variants which recognize epitopes normally expressed by healthy cells (autoantigens). T cells which express autoreactive TCR variants are eliminated during the process of negative selection. The T CD4+ or T CD8+ cells resulting from positive selection migrate to the thymus medulla and interact with autopeptides complexed with MHC expressed at the surface of medullary thymic epithelial cells (mTECs) and dendritic cells. This interaction triggers the process of apoptotic deletion of autoreactive T cells. Negative selection is enabled by the ability of mTECs to express peptides (tissue-restricted antigens (TRAs) which are specific for different tissues of the organism (26, 27, 28). The expression of TRAs in mTEC is regulated by transcription factors, Aire and Fezf2. (C) Differentiation of T CD4+ cells. Following the process of central selection, T cells which do not react with autoantigens migrate to the lymphoid organs (spleen and lymph nodes) and then spread to all peripheral tissues. Depending on the signaling triggered by various cytokines, T CD4+ cells can differentiate to various types of T helper cells (Th) and T regulatory lymphocytes (Tregs) (28). (D) The mechanisms regulating activation and anergy of T lymphocytes. Activation of T lymphocytes: TCR expressed on T lymphocytes recognizes antigen complexed with MHC expressed by antigen-presenting cells (APC). T cell activation is triggered only in the presence of additional stimulation resulting from the interaction of CD28 and CD80/86 receptor. Anergy: In the absence of co-stimulatory signal, T cell undergoes anergy. The signals inhibiting T cell activity are generated by interaction between CTLA-4 and PD-1 receptors (on T cell surface) and their respective ligands (CD80/86 and PD-L1/2), expressed by APC. Inhibition: Tregs inhibit the functioning of T CD4+ and T CD8+ cells by triggering several mechanisms, including secretion of cytokines (interleukin-10 (IL-10), transforming growth factor B (TGFB), and IL-35) leading to inhibition of T cell proliferation, sequestration of IL-2, which triggers T cells’ apoptosis, as well as secretion of granzymes which exert cytotoxic effect on T cells. Tregs can also act directly by the molecules expressed on their cell surface which interact with T cell surface receptors leading to proliferation attenuation (30).

The key mechanism behind AITD development is the loss of immune tolerance to autoantigens. Immune tolerance is the result of processes localized in the thymic and extrathymic tissues (referred as a central and peripheral tolerance, respectively) (Fig. 2). Bone marrow-derived lymphocyte precursors migrate to the thymus to undergo positive selection (in thymus cortex), with the following negative selection (in thymus medulla). The key transcriptional factors involved in maintaining the immune tolerance are Aire and Fezf2, which regulate the expression of tissue-restricted antigens in medullary thymic epithelial cells (mTEC). The mechanisms utilized by both transcription regulators are different. Fezf2 is a classical transcription factor which directly binds to the transcription start sites of TRA genes. In contrast, Aire acts indirectly, by interacting with histone H3 and recruiting proteins which regulate chromatin structure and transcription. It is estimated that expression of more than 60% of TRA genes is controlled by Aire and/or Fezf2; however, the overlap between Fezf2- or Aire-regulated TRA genes is limited. In consequence, both functional Fezf2 and Aire are required for efficient suppression of autoimmune responses. Loss of any of the TRA transcriptional regulators leads to immune disturbances. *AIRE* mutations result in autoimmune polyendocrinopathy syndrome type 1, characterized by a wide spectrum of autoimmunological dysfunctions affecting peripheral endocrine tissues, including the parathyroid and adrenal cortex. In mice, loss of *Fezf2* leads to enhanced autoantibody secretion in serum and elevation of activated T cells in secondary lymphoid organs. So far, no human *FEZF2* mutations were linked with autoimmune disease (26).

mTEC present TRA peptides complexed with major histocompatibility complex (MHC) to the T cells which enables elimination of autoreactive lymphocytes in the process of negative selection. The resulting T CD4+ cells undergo cytokine-regulated differentiation into various types of T helper cells (Th) and T regulatory lymphocytes (Tregs) (26, 27, 28). The autoreactive T cells which still remain following negative selection are removed in the additional processes which include anergy and Treg-mediated inhibition. The anergy is defined as a functional inactivation preventing the lymphocyte from activating an immune reaction against the antigen. The inhibition of T cells is triggered by CTLA-4 and PD-1 receptors which interact with antigen-presenting cells (APC)-expressed CD80/86 and PD-L1/2, respectively (29). The maturation and functioning of Tregs is regulated by Foxp3 transcription factor, which controls the expression of genes crucial for Treg functioning. Tregs inhibit the functioning of T CD4+ and T CD8+ cells by triggering several mechanisms, including secretion or sequestration of cytokines, and via direct interaction leading to attenuation of T cells proliferation (7, 30) (Fig. 2).

**Figure 3:**
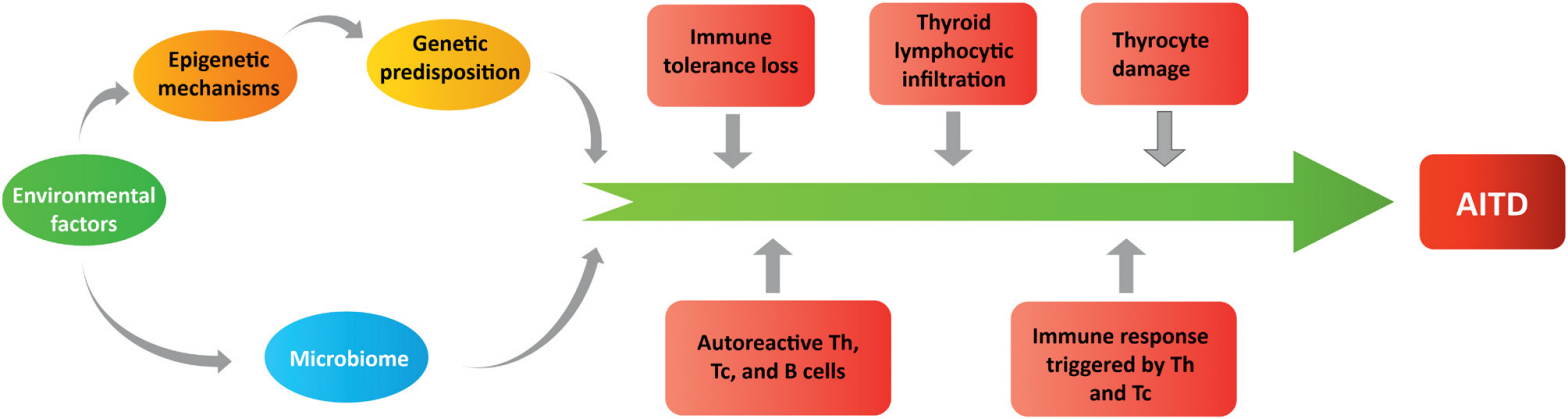
The mechanisms contributing to autoimmune thyroid disease (AITD) pathobiology. The mechanisms triggering the cascade of events leading to AITD involve the interplay between environmental factors (e.g. viral infection), epigenetic/genetic predispositions, and microbiome of which dysfunction contributes to the loss of immune tolerance, activation of autoreactive lymphocytes, and inflammation, leading to the damage of thyrocytes and clinical AITD.

The loss of immune self-tolerance lies in the center of AITD pathobiology (Fig. 3). It may result from the loss of central tolerance (i.e. disturbed deletion of autoreactive T cells in the thymus), dysfunction of peripheral tolerance (i.e. impaired apoptosis of self-reactive T cells and inhibition of the activity of Tregs), and disturbed anergy.

### The mechanisms of immune response in AITD

**Figure 4:**
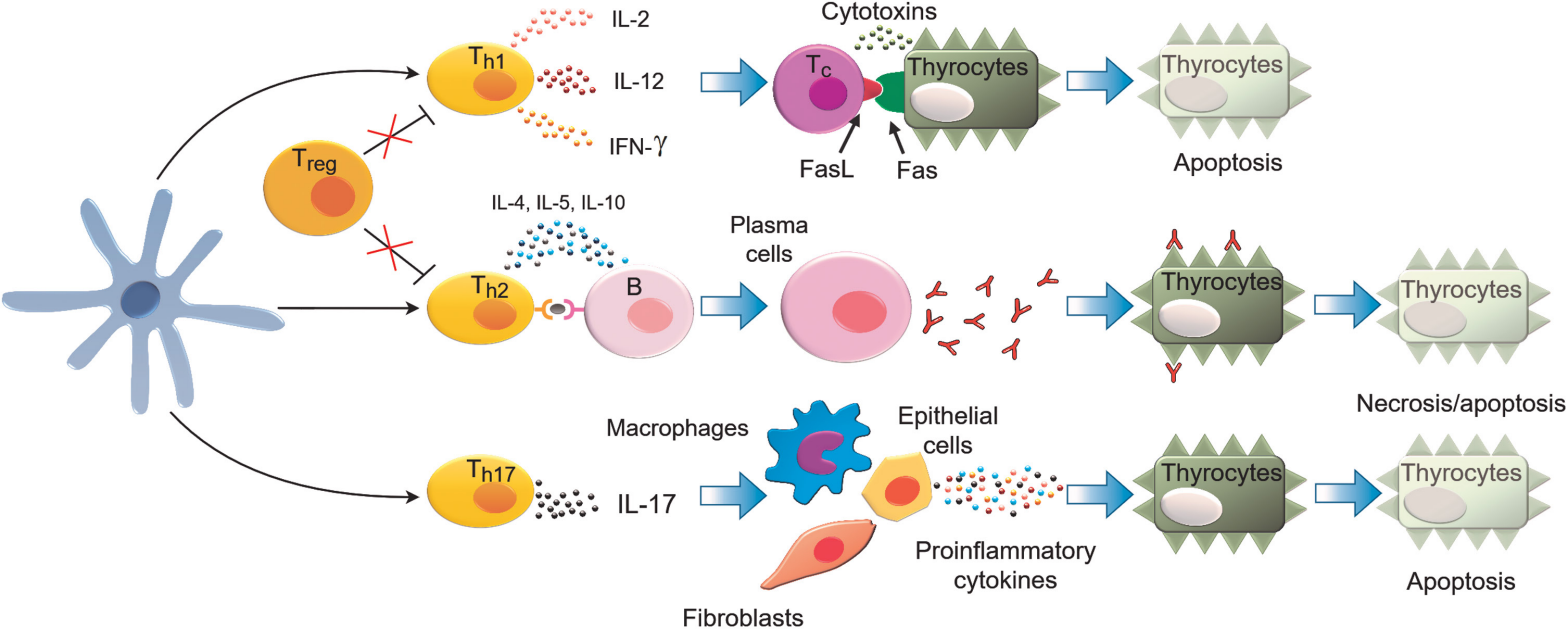
The key mechanisms leading to the destruction of thyroid gland in Hashimoto’s thyroiditis (HT). APC activate T CD4+ lymphocytes which trigger their differentiation into T helper cells (Th1, Th2, and Th17). Th1 lymphocytes secreting mainly IL-12, TNFA, and INFG activate cytotoxic T lymphocytes (Tc) and macrophages which directly target and destroy thyroid follicular cells. The cytokines released by Th1 activate Tc, triggering apoptosis of thyrocytes induced by cytotoxins (perforin, granzymes, and granulysine) or FasL–Fas interaction. Thyroid glands of HT patients express high levels of Fas on the surface of follicular cells. Activated Tc express FasL, which interacts with Fas on thyrocytes, triggering pro-apoptotic signaling cascade. Th2 cells stimulate B cells leading to the formation of plasma cells which produce antibodies directed against thyroid autoantigens which bind thyroid autoantigens and induce thyrocyte apoptosis (mediated by antibody-dependent cytotoxicity or complement activation). Pro-inflammatory Th17 lymphocytes secrete IL-17 which stimulates macrophages, fibroblasts, and epithelial cells to produce cytokines, triggering apoptosis of thyrocytes. The suppressive actions of T regulatory lymphocytes are attenuated in AITD, preventing the counteraction of pro-inflammatory Th17 activity (7, 24).

The key stage in AITD development is the thyroidal accumulation of APCs expressing MHC class II molecules. The infiltration of thyroid by APC (in particular, dendritic cells and macrophages) may be triggered by inflammation resulting from viral or bacterial infection or the exposure of thyroid cells to toxins. In addition, thyroid follicular cells of AITD patients abnormally express interferon-gamma (IFNG)-induced MHC class II molecules which enable the presentation of thyroid autoantigens, facilitating T cells activation (1). In the advanced thyroiditis, thyroid gland is infiltrated by B cells (representing up to 50% of the infiltrating immune cells), as well as cytotoxic T lymphocytes (Tc) and CD4+ cells (7). The interaction with APCs leads to the activation of T CD4+ cells which differentiate into Tregs and Th lymphocytes, including Th1, Th2, and Th17 cells. In HT, Th and Tc trigger the destruction of thyroid gland by inducing several mechanisms involving cytokines and/or cytotoxins (Fig. 4). Notably, AITD is associated with attenuated Tregs which normally counteract pro-inflammatory Th17 activity. Pro-inflammatory Th17 activation and Tregs attenuation are considered as the two key mechanisms contributing to the loss of self-tolerance in autoimmune diseases (7).

Apoptotic loss of thyrocytes in AITD can also be triggered by disruption of thyroxisome (a multiprotein complex including caveolin-1, TPO, and dual oxidase) at the apical thyrocyte membrane, induced by cytokines released by Th1 cells (IFNG, IL1A) or by paracrine- or autocrine-mediated thyrocyte activation. IL1B, of which secretion is increased in HT, activates the expression of Fas and FasL (Fas ligand) at the surface of thyrocytes which enables the activation of apoptosis by thyrocyte–thyrocyte interactions (7). Furthermore, IL1B and TNFA may contribute to upregulation of SphK1 sphingosine kinase in HT, leading to the increased secretion of sphingosine‐1‐phosphate (S1P), which is a ligand of S1PR1, a G protein-coupled receptor on T CD4+ cells (31). Notably, the expression of S1PR1 is increased in T CD4+ cells in autoimmune thyroiditis, triggering the activation of STAT3 signaling through S1PR1/mTOR/PSer^727^STAT3 and S1PR1/JAK2/PTyr^705^STAT3 cascades. In turn, STAT3 recurrently activates the expression of S1PR1, providing the positive feedback regulatory mechanism which ultimately leads to enhanced production of inflammatory cytokines including IL‐17A, IL‐21, IFNG, and IL‐6 (31).

In GD, T cells are activated following the presentation of TSHR peptides. Activated T cells trigger the activation of B cells and plasma cells infiltrating the thyroid to produce autoantibodies directed against TSHR, thereby affecting the secretion of TH. The functioning of B cells and plasma cells is also regulated by liver-produced insulin growth factor 1 (IGF1). The activated T and B cells infiltrating thyroid release pro-inflammatory cytokines, IL-2 and IL-17, which leads to further activation of the immune cells reactive to TSHR (6). IL-17 is secreted by Th cells which can be suppressed by Tregs. It was suggested that activation of inflammatory response in GD could result from depletion of Treg cells expressing CD4+CD25+ and Foxp3 transcription factor. The loss of Treg CD4+CD25+Foxp3+ cells disables suppression of Th1 and Th2 cells, responsible for cytotoxic and antibody-based immune responses, respectively (6).

In contrast to TPO and Tg, TSHR is widely expressed in extrathyroidal tissues and cells, including lymphocytes, adipose tissue, and fibroblasts. In consequence, the presence of TSHR antibodies contributes to the extrathyroidal GD manifestations, such as Graves’ ophthalmopathy (GO), Graves’ dermopathy, or GD-associated thymus hyperplasia (1, 6). TSHR autoantigen is presented by macrophages and B cells recruited to the orbit, which leads to T cell activation (5). In turn, activated T cells initiate immunological attack to the orbital fibroblasts expressing TSHR. In response to the cytokines released by Th cells, orbital fibroblasts produce and deposit large amounts of glycosaminoglycans such as hyaluronan. This in turn leads to the increase of osmotic pressure, followed by water uptake and swelling of the extraorbital muscles. The cytokines released by Th cells stimulate preadipocytes differentiation and adipogenesis contributing to the increased accumulation of orbital adipose tissue. Both fibroblasts and adipocytes express TSHR, which is activated by TSHRAb, thereby stimulating hyaluronan synthesis and adipogenesis. This process is additionally stimulated by IGF1-mediated stimulation of IGF1R expressed by fibroblasts and adipocytes as well as the crosstalk of TSHR and IGF1R signaling pathways which converge in synergistic stimulation of DNA synthesis, proliferation, and hyaluronan secretion (5, 6, 32). Similar molecular pathophysiology may underlie the Graves’ dermopathy (6).

The common mechanism involved in the initiation and acceleration of the inflammatory processes in AITD is the Th1-cytokine/chemokine axis (33). Th1 cells produce IFNG and TNFA which stimulate thyrocytes (in HT and GD) and retroorbital cells in thyroid eye disease to secrete chemokines (CXCL10, CXCL9, and CXCL11). The latter bind and activate CXCR3 receptor on Th1 cells, leading to enhanced IFNG and TNFA release and creating a positive feedback circuit which accelerates recruitment and activation of inflammatory cells in the affected organs. This mechanism is reflected by the elevated levels of CXCL9, CXCL10, and CXCL11 in serum of HT patients. In GD, the CXCL10-mediated recruitment of Th1 cells plays a particular role in the early phase of the disease. In patients with active and relapsing GD, high serum CXCL10 levels are found which decrease after treatment. Those observations suggested that although Th2 cells were initially considered as the predominant modulators of GD autoimmunity, the launch of the GD active phase and relapse are rather shaped by Th1 lymphocytes. Similar findings were reported regarding the active phase of thyroid eye disease, in which elevated serum CXCL10, CXCL9, and CXCL11 levels were found (33).

### Genetic and environmental factors contributing to AITD

It is generally acknowledged that both genetic and environmental factors contribute to AITD development. The key environmental risk factors include smoking, iodine excess, deficiency of selenium and vitamin D, stress, exposure to chemicals, as well as bacterial and viral infection or IFNA treatment (1). The cellular responses to viral infection involve IFNA which induces specific reprogramming of gene expression. In individuals with susceptible genetic background, this mechanism may induce pathological autoimmune reactions (34). In particular, the associations between chronic infection of hepatitis C virus (HCV) and AITD have been repeatedly reported. In general, HCV patients have significantly increased risk to develop hypothyroidism and present TgAb and TPOAb. The proposed mechanism of HCV-induced AITD includes HCV interaction with CD81 receptor on thyroid cells, and induction of IFNG-inducible chemokines which recruit Th1 lymphocytes. The latter secrete IFNG and TNFA, which further stimulate thyrocytes to produce chemokines, resulting in self-feeding circuit accelerating thyroid inflammation and damage (35). The link between genetic susceptibility and environmental factors is provided by epigenetic regulatory mechanisms. Both thyroid tissues and lymphocytes of AITD patients reveal widespread epigenetic modifications affecting chromatin condensation (i.e. DNA methylation and histone modifications) (36) as well as dysregulated expression of ncRNAs.

The most relevant data on the genetic AITD background come from studies involving patients and their families. The heritability of HT and GD has been estimated to 65 and 63%, respectively, with shared genetic effects contributing to 8% of AITD variance (37). The AITD-associated SNPs affect the genes involved in immune responses (e.g. *HLA*, *PTPN22*, *CTLA4*, and *IL2RA*), thyroid function (e.g. *TSHR*, *FOXE1*), as well as other processes (e.g. *LPP*, *TRIB2*) (Supplementary Table 1, see section on supplementary materials given at the end of this article). Remarkably, no TPO polymorphisms were linked with AITD (1). Both GD and HT share several common genes (*HLA*, *PTPN22*, and *CTLA-4*) in which SNPs are linked with increased AITD risk (27). The key genetic association linked with both GD and HT are HLA risk alleles. It is postulated that the HLA-DR SNPs affect the 3D structure of the pocket-binding pathogenic peptides which are presented to T cell receptor (TCR) (2). However, it is estimated that HLA variants represent less than 10% of the AITD genetic background. Furthermore, recent population-based studies revealed that the shared genetic background of HT and GD is limited (37).

**Figure 5:**
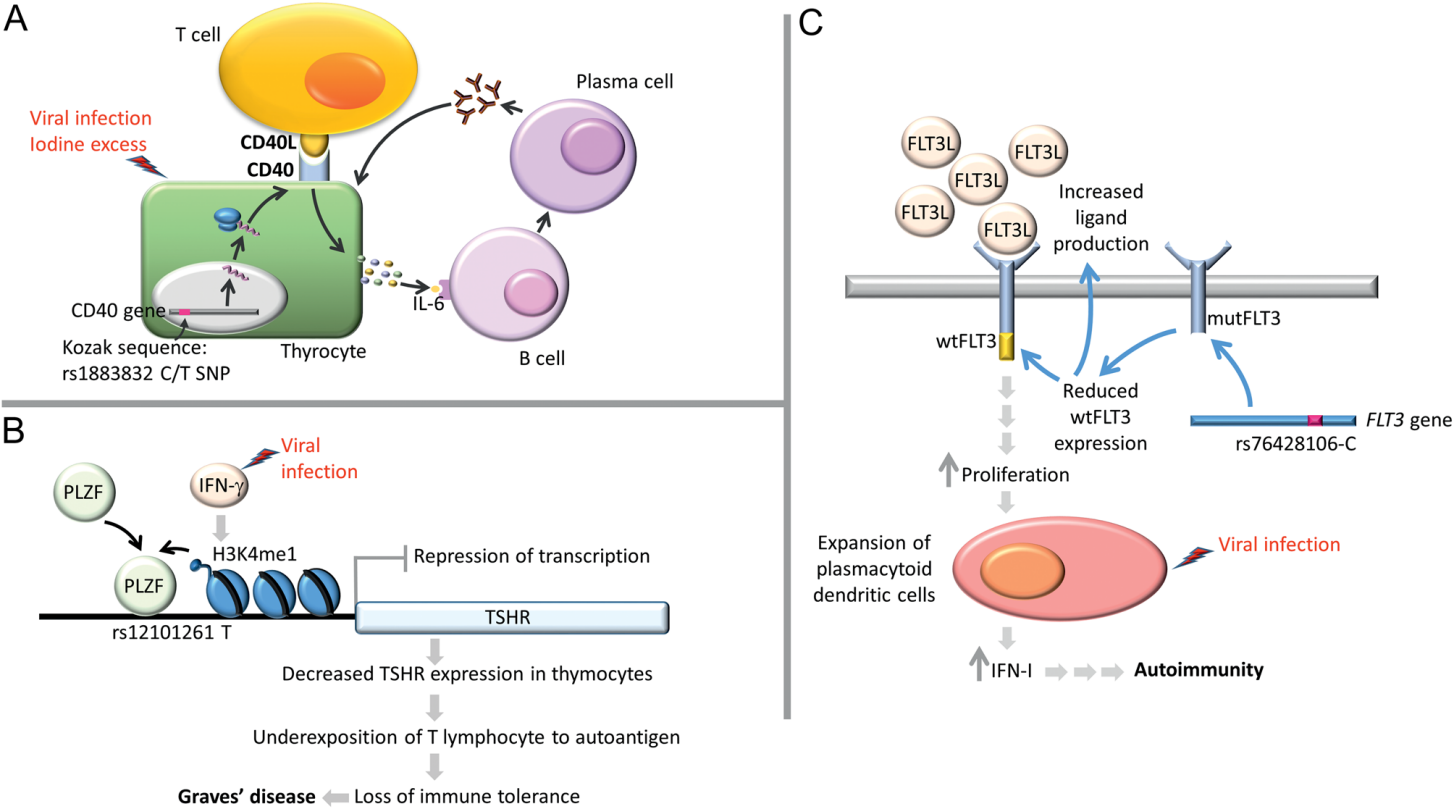
The interference between environmental and genetic factors contributing to AITD development. (A) The GD-associated C allele of CD40 rs1883832 C/T SNP affects Kozak sequence resulting in increased translation efficiency of CD40 receptor. CD40 is expressed on immune cells (e.g. B lymphocytes) and thyrocytes. The environmental factors (e.g. viral infection or iodine excess) lead to the local inflammation, resulting in T cell-mediated CD40 activation, triggering cascades activating the expression of cytokines, including IL-6. The latter acts on B cells, leading to their activation and differentiation into plasma cells which secrete large amounts of thyroid-specific antibodies (40, 62). (B) The GD-associated rs12101261 TSHR SNP affects the binding site of promyelocytic leukemia zinc finger protein (PLZF) transcription factor which acts as a *TSHR* repressor. T allele of rs12101261 determines stronger binding of PLZF, thereby contributing to more efficient repression of *TSHR* transcription. IFNA (e.g. induced by viral infection) induces enrichment of histone H3 lysine 4-monomethylated (H3K4me1) at the site of rs12101261 and augments PLZF-mediated *TSHR* repression. This in turn leads to decreased intrathymic TSHR expression which facilitates loss of immune tolerance by T lymphocytes underexposed to the thyroid autoantigen (34). (C) *FLT3* encodes fms-related tyrosine kinase 3 which acts as a receptor involved in functioning of dendritic cells and responses to viral infections. The HT-linked rs76428106-C allele of *FLT3* creates a cryptic splice site which introduces the premature stop codon, potentially leading to the synthesis of the receptor devoid of the kinase domain. The expression of the rs76428106-C variant is associated with >20% decrease of the WT FLT3 and nearly two-fold increase of the FLT3 ligand. The resulting increase of the FLT3 ligand level leads to overactivation of the full-length receptor, contributing to the preferential expansion of plasmocytoid dendritic cells which in response to viral infection produce large amounts of interferon. The same study also confirmed that this variant was predisposing to other autoimmune diseases (i.e. systemic lupus erythematosus, rheumatoid-factor/anti-CCP-positive rheumatoid arthritis, and coeliac disease) (41).

The mechanisms by which most SNPs in the AITD-linked genes contribute to thyroid autoimmunity are mostly unknown, with a few notable exceptions (Fig. 5). The AITD-related SNPs can influence translation efficiency, transcriptional repression, or alternative splicing of the affected genes (*CD40*, *TSHR*, and *FLT3*). Regarding the failure of immune tolerance to TSHR, two possible mechanisms have been proposed. Brand *et al*. showed that GD-predisposing SNPs localized in intron 1 of *TSHR* (rs179247-AA and rs12101255-TT) are associated with decreased expression ratio of the full-length TSHR (flTSHR) relative to the two TSHR splice variants, ST4 and ST5. The authors suggested that translation of the short ST4 and ST5 transcripts might result in soluble polypeptides which could possibly induce autoimmune response to TSHR (38). This hypothesis was later undermined by the study of Pujol-Borrell/Colobran group who analyzed larger cohort of patients and showed that expression of ST4 and ST5 is not affected by rs179247 variants (neither protective nor predisposing). Furthermore, the rs179247-protective genotype was associated with higher thymic expression of TSHR, without affecting its expression in the thyroid. According to that study, TSHR protein in the thymus may be expressed both as a membrane-bound receptor and as soluble ST4 and ST5 peptides, and that the failure of TSHR tolerance could be the lack of presentation of flTSHR by APCs in thymic medulla (39). The detailed mechanism by which SNPs located in intron 1 of TSHR gene may affect the expression of TSHR was elucidated by Stefan *et al.* (34) (Fig. 5). The environmental factors (e.g. viral infection or iodine excess) provide additional signals which modify the functioning of the affected genes involved in AITD pathology (e.g. by triggering inflammation-induced signaling cascades or epigenetic reprogramming). The final effects of these mechanisms, leading to AITD development, include B cell activation and secretion of thyroid-specific autoantibodies, loss of immune tolerance, or expansion of dendritic cells (Fig. 5) (34, 40, 41).

**Figure 6:**
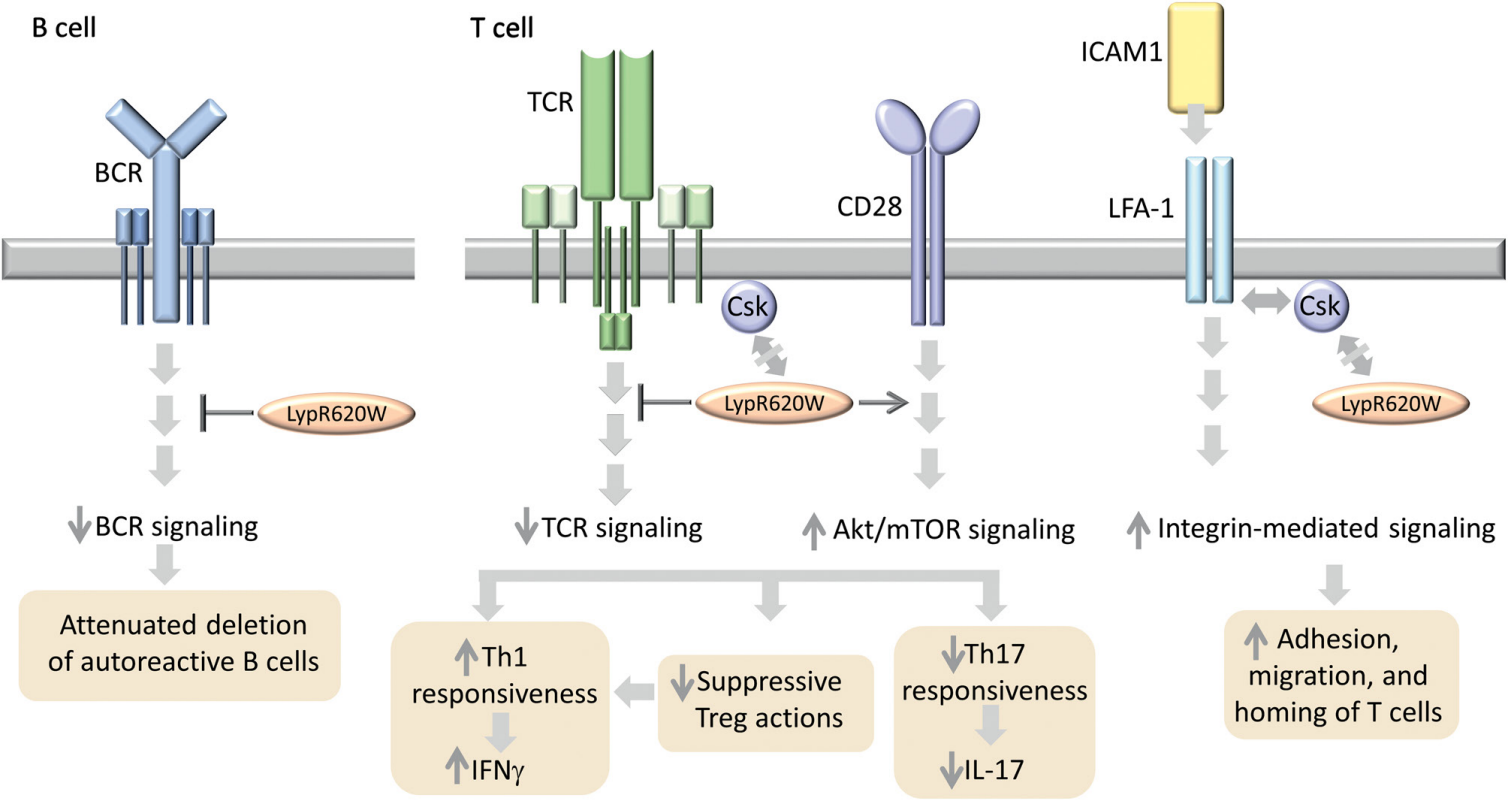
The mechanisms by which *PTPN22* polymorphism may contribute to AITD development. *PTPN22* encodes lymphoid tyrosine phosphatase (Lyp) which dephosphorylates Src family of kinases. WT Lyp affects the functioning of B cells (left panel) and T cells (right panel) by inhibiting the signaling triggered by BCR and TCR, respectively. A 1858C/T SNP leads to R620W substitution, resulting in LypR620W variant which attenuates BCR and TCR signaling more strongly than the WT counterpart. It is proposed that dysfunction of Lyp can lead to inefficient deletion of autoreactive B cells. The WT Lyp is recruited to plasma membrane where it interacts with C-terminal Src kinase (Csk). Following TCR stimulation, Lyp dissociates from Csk and is recruited to lipid rafts to dephosphorylate its substrates. R620W substitution impairs Csk binding, leading enhanced LypR620W recruitment to lipid rafts and stronger inhibition of TCR signaling. In T cells, apart from inhibition of TCR cascade, LypR620W enhances the signaling triggered by CD28, resulting in Akt/mTOR activation. These alterations lead to the attenuation of the suppressive Treg actions which cannot prevent Th1 responsiveness, leading to the increased production of IFNG. On the other hand, the responsiveness of Th17 lymphocytes is reduced, as reflected by decreased IL-17 production. LypR620W mutant contributes to the enhanced adhesion, migration, and homing of T cells by augmenting the signaling induced by lymphocyte function-associated antigen–1 (LFA-1), an integrin receptor regulating the adhesion and migration of T cells. The efficient attenuation of LFA-1 signaling is mediated by Lyp–Csk complexes. Since LypR620W does not interact with Csk, it cannot inhibit LFA-1 signaling (42, 63, 64, 65).

The 1858C/T SNP of *PTPN22* is found in European population patients with AITD and other autoimmune diseases (42). *PTPN22* encodes lymphoid tyrosine phosphatase which dephosphorylates Src family of kinases, leading to the attenuation of TCR and B cell receptor. The proposed models by which 1858C/T SNP variant may contribute to AITD development include impaired regulation of B and T cells functioning (Fig. 6).

As all autoimmune diseases, AITD affects females much more frequently than males. This over-responsiveness of the female immune system is at least partially linked with X chromosome-encoded genes involved in the immune regulation (e.g. *FOXP3*, *CD40L*) and encoding IL receptors (*IL3RA*, *IL9R*, *IL13RA1*, and *IL13RA2*). The hypotheses explaining the female predisposition to autoimmune diseases include skewed X chromosome inactivation, reactivation of genes on silenced X chromosome, and X chromosome monosomy in peripheral lymphocytes (Table 2) (24). According to the recent twin study, although the same genetic factors contribute to HT development in both sexes, their impact on male HT is much stronger than in females. The authors suggested that this could be explained by greater variance of environmental factors in females (37).

**Table 2:** The possible mechanisms behind female predisposition to AITD (24).

Mechanism	Effect on immune system
Immune modulatory effects of estrogens	Estrogens acting on specific receptors expressed in lymphocytes lead, among others, to: 1) enhanced production of antibodies and autoantibodies by B cells; 2) decrease of T CD4+ cells in thymus, and increase of extrathymic T cell lymphopoiesis, resulting in disturbed negative T cell selection; 3) enhanced expression of perforin, IL-10, and TGFB by Tregs.
Skewed X chromosome inactivation	Selective expression of mother-derived or father-derived chromosome in different tissues may lead to T-cells-restricted expression of genes encoded by X chromosome derived from one parent, while other cells and organs (e.g. the thymus) may express genes encoded by the chromosome inherited from another parent. Consequently, T lymphocytes recognize peptides expressed by the other cells as antigens.
Reactivation of genes on silenced X chromosome	Restored functioning of genes involved in immune regulation, which are normally silenced, may lead to over-responsiveness of the immune system.
Chromosomal monosomy	Loss of one of the chromosomes in peripheral B- and/or T cells may lead to deficiency of genes suppressing over-responsiveness of the immune system.
Fetal microchimerism	Fetal cells (including cytotoxic T cells) migrate to mother and trigger immune response.

## The role of non-coding RNA in AITD

**Figure 7:**
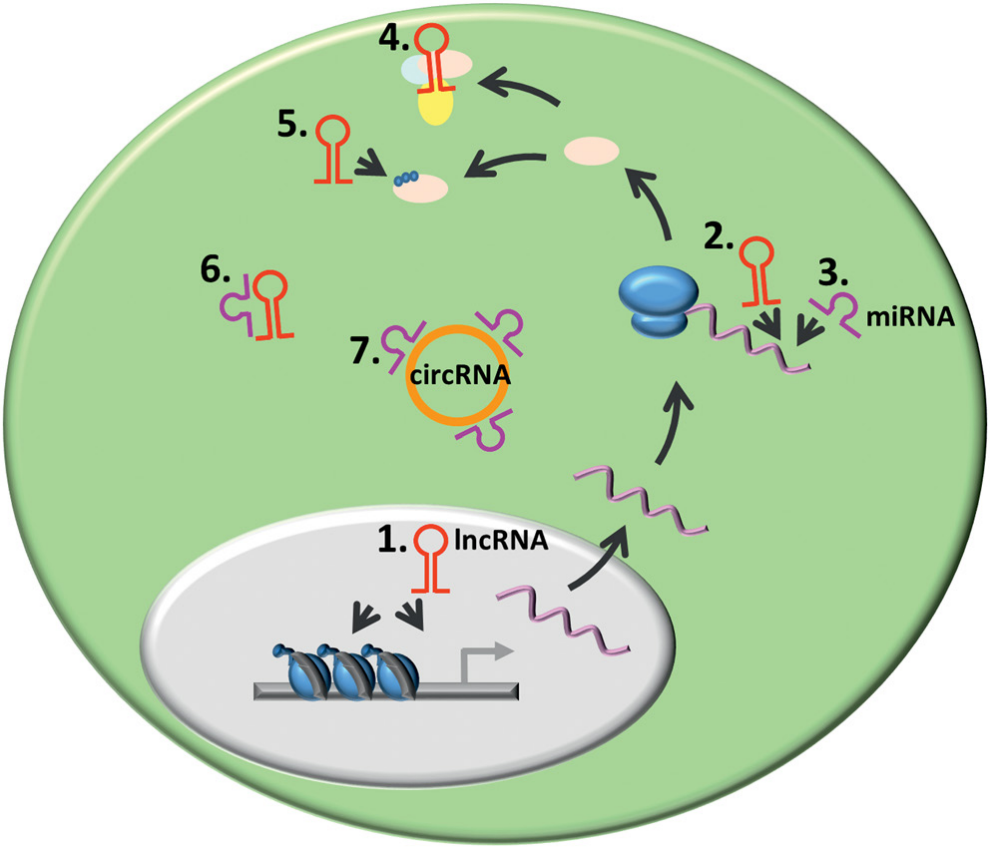
The mechanisms of ncRNA-mediated regulation of gene expression. (1.) LncRNA can affect gene expression by influencing chromatin regulation or the functioning of transcription factors. (2.) LncRNA and (3.) miRNA affect mRNA translation and/or promote mRNA degradation. (4.) LncRNA can act as scaffolds recruiting and binding proteins. (5.) LncRNA can influence posttranslational protein modifications such as phosphorylation. (6.) LncRNA and (7.) circRNA act as sponges binding miRNAs and precluding their regulatory effects.

NcRNAs are defined as untranslated RNA molecules that regulate gene expression. ncRNAs are categorized depending on their length into long ncRNAs (lncRNAs) and short ncRNAs (e.g. miRNA, piRNAs). Based on their function, ncRNAs are classified into infrastructural (e.g. snRNA, snoRNAs, and rRNAs) and regulatory ncRNAs (e.g. miRNA, lncRNAs, piRNAs, and siRNAs) (43). Regardless of their structural differences, all these molecules are capable of regulation of gene expression by several distinct mechanisms (Fig. 7).

The growing interest in the role of ncRNAs in the pathogenesis of AITD comes from their critical role in the regulation of immune responses. Most studies exploring the role of ncRNAs in AITD are focused on miRNAs. Indeed, expression of miRNAs is broadly altered in blood, serum, plasma, and thyroid tissues of AITD patients (Supplementary Table 2). The key miRNAs, repeatedly reported as linked with AITD pathology, include miR-16, miR-21, miR-22, miR-125a, miR-142, miR-146a, miR-146b, miR-155, miR-200a, miR-326, miR-375, miR-431*, and miR-451 (Supplementary Table 2). The suggested clinical relevance of miRNAs comes mainly from their diagnostic potential. Serum/plasma miRNA expression profiles differentiate healthy individuals from GD patients (e.g. multi-miRNAs–based biomarkers such as miR-762/miR-144-3p or miR-210/miR-155/miR-146) and HT patients (e.g. biomarkers consisting of miR-205/miR-20a-3p/miR-375/miR-296/miR-451/miR-500a). miRNAs expression often associates with clinical parameters, such as TPOAb, correlating with miR-21-5p and miR-142-3p in GD patients, or TgAb and TPOAb correlating with miR-326 in HT patients (44, 45). Regarding the prognostic significance, higher miR-21-5p expression associates with a worse prognosis for GD patients, while disturbed expression of miR-155 in serum of GD patients correlates with the extent of goiter (44, 46). It must be noted, however, that although clinically promising, most of these studies require careful validation on independent, large cohorts of AITD patients and healthy control individuals.

miRNAs can influence immune response by regulating the functioning of dendritic cells and various populations of T cells. For instance, miR-146a suppresses dendritic cell apoptosis and cytokine production by targeting of IRAK and TRAF6, the regulators of NFKB signaling cascade (44). Inhibition of miR-125a-5p expression reduced the proportion of Th1 cells and the expression of IFNG in T CD4+ cells (44). Increased expression of miR-142-3p in GD patients results in inhibition of negative regulation of T CD4+C25+ cells proliferation by Tregs (47). Moreover, decreased miR-200a expression in T CD8+ cells of HT patients possibly results in a more significant production of pro-inflammatory Th1 cytokines, contributing to the destruction of thyroid cells (48). In HT patients, peripheral blood mononuclear cells (PBMCs)-elevated miR-326 modified IL-23/IL-23R/Th17 pathway, thereby promoting differentiation of Th17 cells via regulation of ADAM17 (44).

The studies on other types of ncRNAs in AITD are more limited. Regarding lncRNAs, impaired expression of n335641, TCONS-00022357-xloc-010919, and n337845 was found in B cells of GD patients. Computational analysis revealed that these lncRNAs might regulate B cells by changing the expression of TCL1A and SH2D1A, two regulators of B cell proliferation and survival (49). lncRNAs were also postulated as diagnostic biomarkers distinguishing GD and HT patients from healthy controls (44). So far, only one study delineated the role of circRNAs in AITD. Xiong *et al.* reported altered expression of 627 circRNAs in PBMCs of HT patients. In particular, they demonstrated increased expression of circ_0000075, circ_0012152, and circ_0089172, with receiver operating characteristic (ROC) curve area under the curve (AUC) ranging from 0.715 to 0.673, suggestive of their potential diagnostic significance. Furthermore, the study revealed that circ_0089172 acts by sponging of miR-125a-3p, a regulator of IL-23R expression (miRNA sponges are RNA molecules which bind and sequester miRNA, thereby decreasing their inhibitory effect on the expression of target genes) (50).

To summarize, despite extensive work investigating the expression and role of ncRNA in AITD, this topic is still in its infancy. Importantly, the biological significance of altered ncRNAs in AITD remains an open question. Many discoveries also need to be further verified in larger clinical studies. Nevertheless, it is evident that studies focusing on the role of ncRNAs in AITD can increase the understanding of molecular basis of thyroid autoimmunity and might contribute to the development of new diagnostic and therapeutic strategies.

## The role of microbiome in AITD

Human organism is inhabited by over 10^14^ microorganisms (both commensal and pathogenic), collectively referred to as microbiota (Box 1). The most diverse human microbiome is found in the gastrointestinal tract (GIT), which is estimated to be colonized by over 60% of all microorganisms inhabiting human body. The composition of the intestinal microflora is dominated by anaerobic bacteria such as *Bacteroides*, *Bifidobacterium*, *Eubacterium*, *Fusobacterium*, *Ruminococcus* and, to a lesser extent by *Lactobacillus spp*., *Escherichia coli*, *Enterococcus faecalis*, *Bacillus spp*., *Clostridium spp*., and *Streptococcus spp*. The presence of viruses (including *Caudiovirales*, *Podoviridae,* or *Microviridae*) and fungi (the most common: *Candida*, *Cladosporium*, *Cryptococcus,* and *Saccharomyces*) is also observed. Bacterial flora varies depending on the GIT section; the stomach is almost sterile, while the colon has the highest bacteria load. Moreover, it changes during a human’s life, and its shape is influenced by the composition of the mother’s microflora, type of birth, diet, and other environmental and lifestyle factors (51).

**Box 1. Key terms used in microbiome studies.**

ɑ-diversity: diversity of microorganisms’ composition in one sample or environment

β-diversity: diversity of microorganisms’ composition between two samples or environments

Dysbiosis: loss of microflora homeostasis due to a change in its composition and function

Fecal microbiota transplantation: a procedure involving the transplantation of fecal bacteria from a healthy individual to the recipient

Microbiome: the genome of all microorganisms living in a given environment

Microbiota: a group of organisms inhabiting a given environment

Microflora: all microorganisms inhabiting a given environment, e.g. human digestive tract

Prebiotics: nutrients that stimulate the growth of microorganisms

Probiotics: live microorganisms with potential pro-health benefits mainly by modulating gut microbiota and stimulating immune system

Shannon index: index which determines the probability that two individuals (e.g. bacteria species) drawn from the same samples will belong to different species

Synbiotic: dietary supplement containing both probiotics and prebiotics

**Figure 8:**
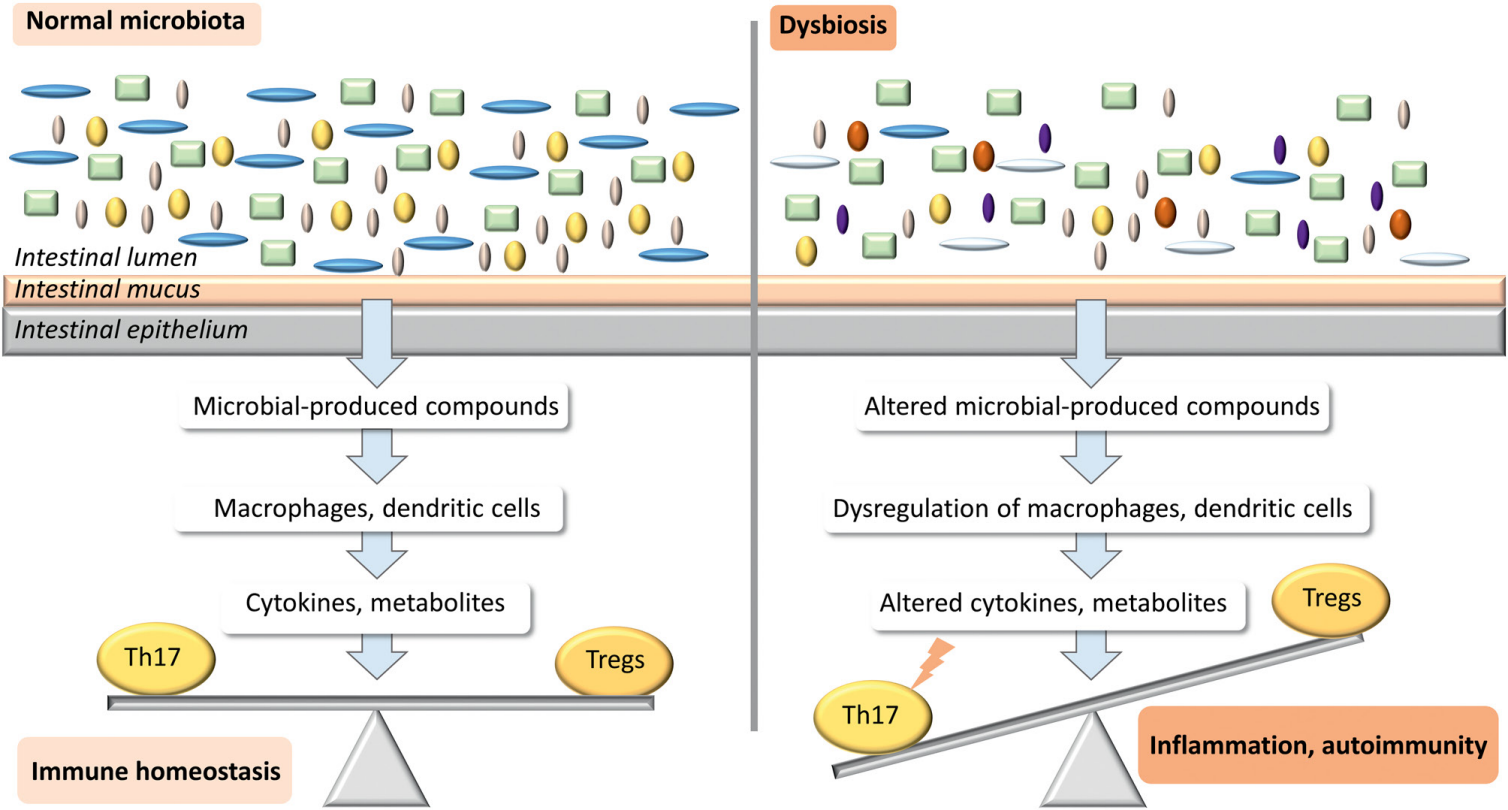
The effects of dysbiosis on immune regulation and autoimmunity. Healthy microbiota produce compounds that act as ligands of TLR and NLR receptors (nucleotide oligomerization domain (NOD)-like receptors) expressed on immune cells, including macrophages and dendritic cells which release cytokines and metabolites such as TGFB, retinoic acid, and interleukins. The cytokines act on target cells, including Th17 lymphocytes and Tregs, ensuring the balance between pro-inflammatory and anti-inflammatory mechanisms. Dysbiosis leads to disturbed regulation of this signaling cascade, shifting the balance toward pro-inflammatory Th17, contributing to inflammation and autoimmunity (51, 53).

The gut microbiome plays several essential physiological functions; it prevents intestine colonization by pathogenic bacteria, facilitates fermentation/degradation of food debris and production of nutrients, and contributes to the development and functioning of gut-associated lymphoid tissue. In particular, the gut microbiome is essential for proper functioning of the immune system. Germ-free mice have reduced Th CD4+ and CD8+, increased Th2 lymphocytes, and attenuated differentiation of Th17 and Treg cells, paralleled by thinner intestinal epithelium mucus layer, reduced intestinal lymphatic tissue, and underdeveloped lymphoid organs (52). The interplay between microbiota and immune regulation involves a complicated network of interactions between specific bacteria, products of their metabolism which target immune cells, as well as cytokines and metabolites which shape the immune homeostasis. The schematic mechanisms involved in microbiota–immune system interactions are presented in Fig. 8. The reader is referred to the recently published more detailed review articles focusing on this topic (51, 53).

Microbiota can influence the functioning of TH, by several mechanisms, including the uptake of micronutrients essential for TH biosynthesis, such as iodine, selenium, and iron. By binding and deconjugating TH metabolites (sulfates and glucuronides), gut bacteria may serve as T4/T3 reservoir. Indeed, supplementation of hypothyroid patients with VSL#3® probiotics (a mixture of four strains of *Lactobacillus spp.*, three strains of *Bifidobacterium spp.*, and *Streptococcus thermophilus*) prevents fluctuations of TH level (54).

The relationship between microbiota dysbiosis and the development of autoimmune diseases such as inflammatory bowel disease, multiple sclerosis, or rheumatoid arthritis is well documented. However, the role of gut microbiome in AITD development is still poorly understood. The first link between microbiota and AITD was reported in 1988, when Penhale and Young showed that the transfer of microbiome from healthy rats to germ-free animals increases the likelihood of AITD development (55). In mice, the composition of gut microbiota contributes to GD susceptibility (56).

The studies on the microbiome of AITD patients clearly show that the composition of the gut microbiota can distinguish between healthy controls, HT, GD, and GO patients, correlate with the stage of disease, level of thyroid autoantibodies, and response to therapy (57) (Supplementary Table 3).

The mechanisms by which microbiome influences AITD development may originate from molecular mimicry resulting from the similarity between microbial and human antigens. The example of such mimicry is the homology between the amino acid sequence of TPO and Tg and protein antigens of *Bifidobacteria* and *Lactobacillus spp*. (58). However, this hypothesis is undermined by studies showing that supplementation of mice with *Lactobacillus rhamnosus* HN001 and *Bifidobacterium lactis* HN019 does not induce the risk of HT development (59). What is particularly important is that probiotics affect the clinical parameters of patients treated with LT4. In hypothyroid patients treated with LT4, synbiotic supplementation for 8 weeks resulted in decrease of TSH concentration, LT4 dose and FT3/TSH ratio (60). Moreover, the results of a large clinical study INDIGO clearly showed the influence of LAB4 probiotics on gut microbiota composition of GD patients and a temporary reduction in the serum level of IgG and IgA antibodies (53). Given these results, we can assume that determining the composition of gut microflora and subsequent use of the appropriate probiotics can contribute to more effective treatment of AITD. The abovementioned studies clearly demonstrate that there is a link between AITD and microbiome. However, the functional consequences of microbiome alterations still require more extensive research, in particular involving patients from different populations.

## Conclusions and perspectives

It is generally acknowledged that AITD is the effect of environmental factors acting on genetically susceptible background with epigenetic mechanisms as mediators. There is strong evidence that polymorphic variants of genes involved in immune regulation or encoding thyroid autoantigens can be linked with AITD predisposition. However, despite multiple studies on molecular AITD background, several important questions still remain to be answered. The mechanisms by which SNPs contribute to AITD development are largely underexplored and require further investigation. It is still unknown what triggers the cascade of processes which lead to the loss of immune tolerance. It remains to be established which of the antigens is recognized as the first in the immune cascade which leads to the dysfunction of thyroid gland. The functional consequences of gut dysbiosis on AITD development and progression remain largely underexplored. In particular, the possible modification of AITD progression by probiotic supplementation remains an open question. Further studies on the molecular and cellular AITD background may help in development of effective novel therapeutic options as illustrated by recently introduced clinical trials on monoclonal antibodies in treatment of GD patients (8, 61).

## Supplementary Material

Supplementary Table S1. Key genetic loci associated with AITD. The table shows genes or non-coding regions at which SNPs predisposing to AITD were found. HT: Hashimoto thyroiditis, GD: Graves’ disease. *Gene locations verified by the authors of this study by accessing https://www.ncbi.nlm.nih.gov/snp/ on March 12<sup>th</sup> 2021.

Supplementary Table S2. Disturbances of non-coding RNAs (ncRNA) expression and functions in autoimmune thyroid disease (AITD). AUC: area under the curve, FFPE: formalin-fixed paraffin-embedded, GD: Graves’ disease, GO: Graves’ ophthalmopathy, HHV: human herpesvirus, HT: Hashimoto’s thyroiditis, lncRNAs: long non-coding RNAs, PBMCs: peripheral blood mononuclear cells, TC: thyroid cancer, TgAb: thyroglobulin autoantibodies, Th: T helper cells, TPOAb: TPO autoantibodies, Treg: T regulatory lymphocyte, TRAb: TSHR autoantibodies, TSLP: thymic stromal lymphopoietin, PTC: papillary thyroid cancer.

Supplementary Table S3. The changes in composition of gut microbiota in AITD patients. Regarding GD, most studies report higher proportion of Bacteroidetes and lower of Firmicutes (at the phyla level) whereas higher number of Bacteroides, Lactobacillus and Prevotella (at the genus level) (Ishaq et al., 2018, Jiang et al., 2021). The abundance of genera such as Blautia, Eubacterium helii_group, Lactobacillus and Dorea distinguished GD patients from healthy controls, suggestive of the potential diagnostic significance. The number of Blautia correlated positively, while Dorea correlated negatively with serum level of TPOAb, indicating clinical significance of these findings (Jiang et al., 2021). Similarly, the abundance of Proteobacteria, Tenericutes and Synergistetes correlated negatively with FT3, FT4, TPOAb and TRAb levels in GD patients (Su et al., 2020). These changes may have biological significance, since Ishaq et al. showed that supernatant of medium used for culturing of B. fragilis (of which proportion is reduced in GD patients) increased the percentage of CD4+ CD25+ FOXP3+ Treg cells and the level of IL-10, while suppressed the percentage of CD4+ IL17+ Th17 cells and the level of IL-17A in PBMCs from healthy controls (Su et al., 2020). The reciprocal links between thyroid function and microbiome are further supported by studies GD patients treated with antithyroid drugs, PTU or methimazole (MMI), which significantly changed the structure of gut microbiota (Sun et al., 2020). In HT patients, key microbiota alterations include increased proportion of gut Firmicutes and reduced number of Bacteroidetes. At the genus level, the abundance of Blautia, Roseburia, Romboutia and Dorea was increased, whereas the number of Faecalibacterium and Prevotella was lower in HT patients when compared with controls (Zhao et al., 2018). The composition of the intestinal microflora also correlates with the clinical parameters of HT patients and response to LT4 treatments. Specifically, the number of Lechnospiraceae, Fusicatenibacter and additional 13 genera were associated with TPOAb level (Zhao et al., 2018), while the proportion of Clostridium coccoides correlated with TSH level and the time of HT disease duration. The abundance of Bacteroides, Faecalibacterium, Prevotella and additional 7 genera was suggested as a promising biomarker distinguishing HT patients from healthy controls (Zhao et al., 2018).

## Declaration of interest

A P-W is a member of European Thyroid Journal Editorial Board; she was not involved in the review process of the manuscript. The other authors declare no conflict of interest.

## Funding

The authors are financially supported by 501-1-025-01-20 CMKP grant.

## References

[1] Mclachlan SM Rapoport B. Breaking tolerance to thyroid antigens: changing concepts in thyroid autoimmunity. Endocrine Reviews 2014 35 59–105. (10.1210/er.2013-1055) 24091783 PMC3895862

[2] Tomer Y Huber A. The etiology of autoimmune thyroid disease: a story of genes and environment. Journal of Autoimmunity 2009 32 231–239. (10.1016/j.jaut.2009.02.007) 19307103 PMC3561494

[3] Wood LC Ingbar SH. Hypothyroidism as a late sequela in patient with Graves’ disease treated with antithyroid agents. Journal of Clinical Investigation 1979 64 1429–1436. (10.1172/JCI109601) 91625 PMC371292

[4] Eckstein A Philipp S Goertz G Banga JP Berchner-Pfannschmidt U. Lessons from mouse models of Graves’ disease. Endocrine 2020 68 265–270. (10.1007/s12020-020-02311-7) 32399893 PMC7266836

[5] Wiersinga WM Advances in treatment of active, moderate-to-severe Graves’ ophthalmopathy. Lancet: Diabetes and Endocrinology 2017 5 134–142. (10.1016/S2213-8587(1630046-8) 27346786

[6] Davies TF Andersen S Latif R Nagayama Y Barbesino G Brito M Eckstein AK Stagnaro-Green A Kahaly GJ. Graves’ disease. Nature Reviews: Disease Primers 2020 6 52. (10.1038/s41572-020-0184-y) 32616746

[7] Wiersinga WM Hashimoto’s thyroiditis. In Thyroid Diseases: Pathogenesis, Diagnosis, and Treatment. Eds Vitti P Hegedüs L. Cham: Springer International Publishing, 2018. (10.1007/978-3-319-45013-1)

[8] Lane LC Cheetham TD Perros P Pearce SHS. New therapeutic horizons for Graves’ hyperthyroidism. Endocrine Reviews 2020 41 873–884. (10.1210/endrev/bnaa022) PMC7567404 32845332

[9] Kahaly GJ Diana T Olivo PD. Tsh receptor antibodies: relevance & utility. Endocrine Practice 2020 26 97–106. (10.4158/EP-2019-0363) 32022598

[10] Morshed SA Ando T Latif R Davies TF. Neutral antibodies to the TSH receptor are present in Graves’ disease and regulate selective signaling cascades. Endocrinology 2010 151 5537–5549. (10.1210/en.2010-0424) 20844004 PMC2954721

[11] Sanders J Miguel RN Bolton J Bhardwaja A Sanders P Nakatake N Evans M Furmaniak J Smith BR. Molecular interactions between the TSH receptor and a thyroid-stimulating monoclonal autoantibody. Thyroid 2007 17 699–706. (10.1089/thy.2007.0041) 17725428

[12] Sanders P Young S Sanders J Kabelis K Baker S Sullivan A Evans M Clark J Wilmot J Hu X Crystal structure of the TSH receptor (TSHR) bound to a blocking-type TSHR autoantibody. Journal of Molecular Endocrinology 2011 46 81–99. (10.1530/JME-10-0127) 21247981

[13] Godlewska M Banga PJ. Thyroid peroxidase as a dual active site enzyme: focus on biosynthesis, hormonogenesis and thyroid disorders of autoimmunity and cancer. Biochimie 2019 160 34–45. (10.1016/j.biochi.2019.02.003) 30742860

[14] Le SN Porebski BT Mccoey J Fodor J Riley B Godlewska M Gora M Czarnocka B Banga JP Hoke DE Modelling of thyroid peroxidase reveals insights into its enzyme function and autoantigenicity. PLoS ONE 2015 10 e0142615. (10.1371/journal.pone.0142615) PMC4666655 26623656

[15] Godlewska M Gawel D Buckle AM Banga JP. Thyroid peroxidase revisited – what’s new? Hormone and Metabolic Research 2019 51 765–769. (10.1055/a-1057-9469) 31826271

[16] Czarnocka B Eschler DC Godlewska M Tomer Y. Chapter 44 – Thyroid autoantibodies: thyroid peroxidase and thyroglobulin antibodies. In Autoantibodies, 3rd ed. Eds Shoenfeld Y Meroni PL Gershwin ME. San Diego: Elsevier, 2014. (10.1016/C2010-0-68545-2)

[17] Godlewska M Czarnocka B Gora M. Localization of key amino acid residues in the dominant conformational epitopes on thyroid peroxidase recognized by mouse monoclonal antibodies. Autoimmunity 2012 45 476–484. (10.3109/08916934.2012.682667) 22559245

[18] Dubska M Banga JP Plochocka D Hoser G Kemp EH Sutton BJ Gardas A Gora M. Structural insights into autoreactive determinants in thyroid peroxidase composed of discontinuous and multiple key contact amino acid residues contributing to epitopes recognized by patients’ autoantibodies. Endocrinology 2006 147 5995–6003. (10.1210/en.2006-0912) 16959834

[19] Jaume JC Burek CL Hoffman WH Rose NR Mclachlan SM Rapoport B. Thyroid peroxidase autoantibody epitopic ‘fingerprints’ in juvenile Hashimoto’s thyroiditis: evidence for conservation over time and in families. Clinical and Experimental Immunology 1996 104 115–123. (10.1046/j.1365-2249.1996.d01-659.x) 8603516 PMC2200393

[20] Williams DE Le SN Hoke DE Chandler PG Gora M Godlewska M Banga JP Buckle AM. Structural studies of thyroid peroxidase show the monomer interacting with autoantibodies in thyroid autoimmune disease. Endocrinology 2020 161 bqaa016. (10.1210/endocr/bqaa016) 32022847

[21] Di Jeso B Arvan P. Thyroglobulin from molecular and cellular biology to clinical endocrinology. Endocrine Reviews 2016 37 2–36. (10.1210/er.2015-1090) 26595189 PMC4740344

[22] Coscia F Taler-Vercic A Chang VT Sinn L O’Reilly FJ Izore T Renko M Berger I Rappsilber J Turk D The structure of human thyroglobulin. Nature 2020 578 627–630. (10.1038/s41586-020-1995-4) 32025030 PMC7170718

[23] Mclachlan SM Rapoport B. Thyroid autoantibodies display both ‘original antigenic sin’ and epitope spreading. Frontiers in Immunology 2017 8 1845. (10.3389/fimmu.2017.01845) PMC5742354 29326719

[24] Frohlich E Wahl R. Thyroid autoimmunity: role of anti-thyroid antibodies in thyroid and extra-thyroidal diseases. Frontiers in Immunology 2017 8 521. (10.3389/fimmu.2017.00521) PMC5422478 28536577

[25] Eleftheriadou AM Mehl S Renko K Kasim RH Schaefer JA Minich WB Schomburg L. Re-visiting autoimmunity to sodium-iodide symporter and pendrin in thyroid disease. European Journal of Endocrinology 2020 183 571–580. (10.1530/EJE-20-0566) 33055303

[26] Takaba H Takayanagi H. The mechanisms of T cell selection in the Thymus. Trends in Immunology 2017 38 805–816. (10.1016/j.it.2017.07.010) 28830733

[27] Simmonds MJ GWAS in autoimmune thyroid disease: redefining our understanding of pathogenesis. Nature Reviews: Endocrinology 2013 9 277–287. (10.1038/nrendo.2013.56) 23529038

[28] Pyzik A Grywalska E Matyjaszek-Matuszek B Rolinski J. Immune disorders in Hashimoto’s thyroiditis: what do we know so far? Journal of Immunology Research 2015 2015 979167. (10.1155/2015/979167) 26000316 PMC4426893

[29] Walker LS Abbas AK. The enemy within: keeping self-reactive T cells at bay in the periphery. Nature Reviews: Immunology 2002 2 11–19. (10.1038/nri701) 11908514

[30] Shevach EM Mechanisms of foxp3+ T regulatory cell-mediated suppression. Immunity 2009 30 636–645. (10.1016/j.immuni.2009.04.010) 19464986

[31] Han C He X Xia X Guo J Liu A Liu X Wang X Li C Peng S Zhao W Sphk1/S1P/S1PR1 signaling is involved in the development of autoimmune thyroiditis in patients and NOD.H-2(h4) mice. Thyroid 2019 29 700–713. (10.1089/thy.2018.0065) 30963819

[32] Neumann S Krieger CC Gershengorn MC. Targeting TSH and IGF-1 receptors to treat thyroid eye disease. European Thyroid Journal 2020 9 59–65. (10.1159/000511538) 33511086 PMC7802449

[33] Fallahi P Ferrari SM Ragusa F Ruffilli I Elia G Paparo SR Antonelli A. Th1 chemokines in autoimmune endocrine disorders. Journal of Clinical Endocrinology and Metabolism 2020 105 dgz289. (10.1210/clinem/dgz289) 31863667

[34] Stefan M Jacobson EM Huber AK Greenberg DA Li CW Skrabanek L Conception E Fadlalla M Ho K Tomer Y. Novel variant of thyroglobulin promoter triggers thyroid autoimmunity through an epigenetic interferon alpha-modulated mechanism. Journal of Biological Chemistry 2011 286 31168–31179. (10.1074/jbc.M111.247510) 21757724 PMC3173071

[35] Colaci M Malatino L Antonelli A Fallahi P Giuggioli D Ferri C. Endocrine disorders associated with hepatitis C virus chronic infection. Reviews in Endocrine and Metabolic Disorders 2018 19 397–403. (10.1007/s11154-018-9475-y) 30499080

[36] Wang B Shao X Song R Xu D Zhang JA. The emerging role of epigenetics in autoimmune thyroid diseases. Frontiers in Immunology 2017 8 396. (10.3389/fimmu.2017.00396) PMC5383710 28439272

[37] Skov J Calissendorff J Eriksson D Magnusson P Kampe O Bensing S Kuja-Halkola R. Limited genetic overlap between overt Hashimoto’s thyroiditis and Graves’ disease in twins: a population-based study. Journal of Clinical Endocrinology and Metabolism 2021 106 1101–1110. (10.1210/clinem/dgaa956) 33382429 PMC7993582

[38] Brand OJ Barrett JC Simmonds MJ Newby PR Mccabe CJ Bruce CK Kysela B Carr-Smith JD Brix T Hunt PJ Association of the thyroid stimulating hormone receptor gene (TSHR) with Graves’ disease. Human Molecular Genetics 2009 18 1704–1713. (10.1093/hmg/ddp087) 19244275

[39] Marin-Sanchez A Alvarez-Sierra D Gonzalez O Lucas-Martin A Selles-Sanchez A Rudilla F Enrich E Colobran R Pujol-Borrell R. Regulation of TSHR expression in the thyroid and Thymus May contribute to TSHR tolerance failure in Graves’ disease patients via two distinct mechanisms. Frontiers in Immunology 2019 10 1695. (10.3389/fimmu.2019.01695) PMC6657650 31379878

[40] Huber AK Finkelman FD Li CW Concepcion E Smith E Jacobson E Latif R Keddache M Zhang W Tomer Y. Genetically driven target tissue overexpression of CD40: a novel mechanism in autoimmune disease. Journal of Immunology 2012 189 3043–3053. (10.4049/jimmunol.1200311) PMC3436983 22888137

[41] Saevarsdottir S Olafsdottir TA Ivarsdottir EV Halldorsson GH Gunnarsdottir K Sigurdsson A Johannesson A Sigurdsson JK Juliusdottir T Lund SH FLT3 stop mutation increases FLT3 ligand level and risk of autoimmune thyroid disease. Nature 2020 584 619–623. (10.1038/s41586-020-2436-0) 32581359

[42] Vang T Nielsen J Burn GL. A switch-variant model integrates the functions of an autoimmune variant of the phosphatase PTPN22. Science Signaling 2018 11 eaat0936. (10.1126/scisignal.aat0936) 29666305

[43] Yin L Zeng C Yao J Shen J. Emerging roles for noncoding RNAs in autoimmune thyroid disease. Endocrinology 2020 161 bqaa053. (10.1210/endocr/bqaa053) 32270194

[44] Taheri M Eghtedarian R Dinger ME Ghafouri-Fard S. Dysregulation of non-coding RNAs in autoimmune thyroid disease. Experimental and Molecular Pathology 2020 117 104527. (10.1016/j.yexmp.2020.104527) 32916160

[45] Liu Y Cui X Wang S Liu J Zhao N Huang M Qin J Li Y Shan Z Teng W. Elevated MicroRNA-326 levels regulate the IL-23/IL-23R/Th17 cell axis in Hashimoto’s thyroiditis by targeting a disintegrin and metalloprotease 17. Thyroid 2020 30 1327–1337. (10.1089/thy.2019.0552) 32204685

[46] Zheng L Zhuang C Wang X Ming L. Serum miR-146a, miR-155, and miR-210 as potential markers of Graves’ disease. Journal of Clinical Laboratory Analysis 2018 32 e22266. (10.1002/jcla.22266) PMC6817012 28569050

[47] Martinez-Hernandez R Sampedro-Nunez M Serrano-Somavilla A Ramos-Levi AM De La Fuente H Trivino JC Sanz-Garcia A Sanchez-Madrid F Marazuela M. A microRNA signature for evaluation of risk and severity of autoimmune thyroid diseases. Journal of Clinical Endocrinology and Metabolism 2018 103 1139–1150. (10.1210/jc.2017-02318) 29325052

[48] Bernecker C Lenz L Ostapczuk MS Schinner S Willenberg H Ehlers M Vordenbaumen S Feldkamp J Schott M. MicroRNAs miR-146a1, miR-155_2, and miR-200a1 are regulated in autoimmune thyroid diseases. Thyroid 2012 22 1294–1295. (10.1089/thy.2012.0277) 22957494

[49] Jiang X Wang Y Li X He L Yang Q Wang W Liu J Zha B. Microarray profile of B cells from Graves’ disease patients reveals biomarkers of proliferation. Endocrine Connections 2020 9 405–417. (10.1530/EC-20-0045) 32432440 PMC7274554

[50] Xiong S Peng H Ding X Wang X Wang L Wu C Wang S Xu H Liu Y. Circular RNA expression profiling and the potential role of hsa_circ_0089172 in Hashimoto’s thyroiditis via sponging miR125a-3p. Molecular Therapy: Nucleic Acids 2019 17 38–48. (10.1016/j.omtn.2019.05.004) 31207490 PMC6579753

[51] Zheng D Liwinski T Elinav E. Interaction between microbiota and immunity in health and disease. Cell Research 2020 30 492–506. (10.1038/s41422-020-0332-7) 32433595 PMC7264227

[52] Round JL Mazmanian SK. The gut microbiota shapes intestinal immune responses during health and disease. Nature Reviews: Immunology 2009 9 313–323. (10.1038/nri2515) PMC4095778 19343057

[53] Masetti G Ludgate M. Microbiome and Graves’ orbitopathy. European Thyroid Journal 2020 9 78–85. (10.1159/000512255) 33511088 PMC7802434

[54] Frohlich E Wahl R. Microbiota and thyroid interaction in health and disease. Trends in Endocrinology and Metabolism 2019 30 479–490. (10.1016/j.tem.2019.05.008) 31257166

[55] Penhale WJ Young PR. The influence of the normal microbial flora on the susceptibility of rats to experimental autoimmune thyroiditis. Clinical and Experimental Immunology 1988 72 288–292. 2970354 PMC1541533

[56] Moshkelgosha S Masetti G Berchner-Pfannschmidt U Verhasselt HL Horstmann M Diaz-Cano S Noble A Edelman B Covelli D Plummer S Gut microbiome in BALB/c and C57BL/6J mice undergoing experimental thyroid autoimmunity associate with differences in immunological responses and thyroid function. Hormone and Metabolic Research 2018 50 932–941. (10.1055/a-0653-3766) 30107619

[57] Virili C Stramazzo I Centanni M. Gut microbiome and thyroid autoimmunity. Best Practice and Research: Clinical Endocrinology and Metabolism 2021 35 101506. (10.1016/j.beem.2021.101506) 33648848

[58] Kiseleva EP Mikhailopulo KI Sviridov OV Novik GI Knirel YA Szwajcer Dey E. The role of components of Bifidobacterium and Lactobacillus in pathogenesis and serologic diagnosis of autoimmune thyroid diseases. Beneficial Microbes 2011 2 139–154. (doi:10.3920/BM2010.00110029 21831795 10.3920/BM2010.0011

[59] Zhou JS Gill HS. Immunostimulatory probiotic Lactobacillus rhamnosus HN001 and Bifidobacterium lactis HN019 do not induce pathological inflammation in mouse model of experimental autoimmune thyroiditis. International Journal of Food Microbiology 2005 103 97–104. (10.1016/j.ijfoodmicro.2004.11.031) 16084270

[60] Talebi S Karimifar M Heidari Z Mohammadi H Askari G. The effects of synbiotic supplementation on thyroid function and inflammation in hypothyroid patients: a randomized, doubleblind, placebocontrolled trial. Complementary Therapies in Medicine 2020 48 102234. (10.1016/j.ctim.2019.102234) 31987229

[61] Kahaly GJ Stan MN Frommer L Gergely P Colin L Amer A Schuhmann I Espie P Rush JS Basson C A novel anti-CD40 monoclonal antibody, Iscalimab, for control of graves hyperthyroidism-A proof-of-concept trial. Journal of Clinical Endocrinology and Metabolism 2020 105 dgz013. (10.1210/clinem/dgz013) 31512728

[62] Jacobson EM Concepcion E Oashi T Tomer Y. A Graves’ disease-associated Kozak sequence single-nucleotide polymorphism enhances the efficiency of CD40 gene translation: a case for translational pathophysiology. Endocrinology 2005 146 2684–2691. (10.1210/en.2004-1617) 15731360

[63] Vang T Landskron J Viken MK Oberprieler N Torgersen KM Mustelin T Tasken K Tautz L Rickert RC Lie BA. The autoimmune-predisposing variant of lymphoid tyrosine phosphatase favors T helper 1 responses. Human Immunology 2013 74 574–585. (10.1016/j.humimm.2012.12.017) 23333624 PMC4011173

[64] Vang T Liu WH Delacroix L Wu S Vasile S Dahl R Yang L Musumeci L Francis D Landskron J LYP inhibits T-cell activation when dissociated from CSK. Nature Chemical Biology 2012 8 437–446. (10.1038/nchembio.916) 22426112 PMC3329573

[65] Burn GL Cornish GH Potrzebowska K Samuelsson M Griffie J Minoughan S Yates M Ashdown G Pernodet N Morrison VL Superresolution imaging of the cytoplasmic phosphatase PTPN22 links integrin-mediated T cell adhesion with autoimmunity. Science Signaling 2016 9 ra99. (10.1126/scisignal.aaf2195) 27703032

