# Supplementary material for: Cellular and molecular basis of thyroid autoimmunity
Source: Eur Thyroid J. 2021 Nov 1;11(1):e210024. doi: 10.1530/ETJ-21-0024 (PMC9142813; doi:10.1530/ETJ-21-0024)
Supplement: Supplementary Table S1. Key genetic loci associated with AITD. The table shows genes or non-coding regions at which SNPs predisposing to AITD were found. HT: Hashimoto thyroiditis, GD: Graves’ disease. *Gene locations verified by the authors of this study by accessing https://www.ncbi.nlm.nih.gov/sn [file supplementary_table_1.pdf]

Supplementary Table S1. Key genetic loci associated with AITD. The table shows genes or non-coding regions at which SNPs predisposing to AITD were found. HT: Hashimoto thyroiditis, GD: Graves' disease. *Gene locations verified by the authors of this study by accessing https://www.ncbi.nlm.nih.gov/snp/ on March 12th 2021.

Gene	Polymorphism	Effect	References
ABO	rs505922	Increased predisposition to GD	(Zhao et al., 2013, Zhang et al., 2020)
BACH2	rs2474619	Increased predisposition to HT and GD	(Zhang et al., 2020)
C14orf177/VRK1	rs1456988	Increased predisposition to GD	(Zhang et al., 2020)
C1QTNF6-RAC2	rs229527 and rs2284038	Increased predisposition to GD	(Zhao et al., 2013, Zhang et al., 2020)
CD40	rs1883832	Increased predisposition to GD	(Kurylowicz et al., 2005, Zhang et al., 2020)
CTLA4	rs1024161	Increased predisposition to GD and HT	(Chu et al., 2011, Zhang et al., 2020)
CTLA4	rs231775	Increased predisposition to GD and HT	(Chen et al., 2018, Fathima et al., 2019)
ESR2	rs4986938	Increased predisposition to GD	(Kisiel et al., 2008)
FAM76B/SESN3	rs12575636	Increased predisposition to HT and GD	(Zhang et al., 2020)
FCRL3	rs17676303	Increased predisposition to GD (especially in Asian)	(Khong et al., 2016)
FCRL3	rs7528684	Increased predisposition to GD	(Zhang et al., 2020)
FOXP3	rs3761548 and rs3761549	Increased predisposition to GD (especially in Asian)	(Li et al., 2020)
GPR174/ITM2A	rs5912838	Increased predisposition to HT and GD	(Zhao et al., 2013, Zhang et al., 2020)
HLA-B	rs1521	Increased predisposition to HT and GD	(Zhang et al., 2020, Zhao et al., 2013)
HLA-DPB1	rs2281388	Increased predisposition to GD	(Zhang et al., 2020)
IL-17F	rs9463772	Increased predisposition to GD	(Guo et al., 2013)
IL-23A	rs11171806	Increased predisposition to GD	(Jia et al., 2015)
IL6	rs1800795	Increased predisposition to HT	(Duraes et al., 2014)

LPP	rs13093110	Increased predisposition to HT and GD	(Zhang et al., 2020)
PD-L1	rs822339, rs2282055, and rs1411262	Increased predisposition to GD	(Mitchell et al., 2009)
PTPN22/LYP	rs2476601	Increased predisposition to GD	(Velaga et al., 2004, Wawrusiewicz-Kurylonek et al., 2019)
RHOH/CHRNA9	rs6832151	Increased predisposition to HT and GD	(Zhang et al., 2020)
RNASET2	rs9355610	Increased predisposition to HT and GD	(Zhang et al., 2020)
SLAMF6	rs1265883	Increased predisposition to HT and GD	(Zhang et al., 2020, Zhao et al., 2013)
TG	rs2294025 and rs4736437	Increased predisposition to GD	(Chu et al., 2011, Zhao et al., 2013, Zhang et al., 2020)
TNFA	rs1800629, rs1800630, and rs1799964	Increased predisposition to GD	(Li et al., 2008)
TNFA	rs1800629	Increased predisposition to HT and GD	(Duraes et al., 2014)
TRIB2	rs1881145	Increased predisposition to HT and GD	(Zhang et al., 2020)
TSHR	rs179247 and rs12101255	Increased predisposition to GD	(Brand et al., 2009, Xiong et al., 2016)
TSHR	rs12101261	Increased predisposition to GD	(Brand et al., 2009, Zhang et al., 2020)
TSHR	rs3783938	Increased predisposition to GD	(Liu et al., 2012)
VDR	TT subtype of the TaqI polymorphism	Increased predisposition to GD	(Veneti et al., 2019)

References:

BRAND, O. J., BARRETT, J. C., SIMMONDS, M. J., NEWBY, P. R., MCCABE, C. J., BRUCE, C. K., KYSELA, B., CARR-SMITH, J. D., BRIX, T., HUNT, P. J., WIERSINGA, W. M., HEGEDUS, L., CONNELL, J., WASS, J. A., FRANKLYN, J. A., WEETMAN, A. P., HEWARD, J. M. & GOUGH, S. C. 2009. Association of the thyroid stimulating hormone receptor gene (TSHR) with Graves' disease. *Hum Mol Genet,* 18**,** 1704-13.

CHEN, X., HU, Z., LIU, M., LI, H., LIANG, C., LI, W., BAO, L., CHEN, M. & WU, G. 2018. Correlation between CTLA-4 and CD40 gene polymorphisms and their interaction in graves' disease in a Chinese Han population. *BMC Med Genet,* 19**,** 171.

CHU, X., PAN, C. M., ZHAO, S. X., LIANG, J., GAO, G. Q., ZHANG, X. M., YUAN, G. Y., LI, C. G., XUE, L. Q., SHEN, M., LIU, W., XIE, F., YANG, S. Y., WANG, H. F., SHI, J. Y., SUN, W. W., DU, W. H., ZUO, C. L., SHI, J. X., LIU, B. L., GUO, C. C., ZHAN, M., GU, Z. H., ZHANG, X. N., SUN, F., WANG, Z. Q.,

SONG, Z. Y., ZOU, C. Y., SUN, W. H., GUO, T., CAO, H. M., MA, J. H., HAN, B., LI, P., JIANG, H., HUANG, Q. H., LIANG, L., LIU, L. B., CHEN, G., SU, Q., PENG, Y. D., ZHAO, J. J., NING, G., CHEN, Z., CHEN, J. L., CHEN, S. J., HUANG, W., SONG, H. D. & CHINA CONSORTIUM FOR GENETICS OF AUTOIMMUNE THYROID, D. 2011. A genome-wide association study identifies two new risk loci for Graves' disease. *Nat Genet,* 43**,** 897-901.

DURAES, C., MOREIRA, C. S., ALVELOS, I., MENDES, A., SANTOS, L. R., MACHADO, J. C., MELO, M., ESTEVES, C., NEVES, C., SOBRINHO-SIMOES, M. & SOARES, P. 2014. Polymorphisms in the TNFA and IL6 genes represent risk factors for autoimmune thyroid disease. *PLoS One,* 9**,** e105492.

FATHIMA, N., NARNE, P. & ISHAQ, M. 2019. Association and gene-gene interaction analyses for polymorphic variants in CTLA-4 and FOXP3 genes: role in susceptibility to autoimmune thyroid disease. *Endocrine,* 64**,** 591-604.

GUO, T., HUO, Y., ZHU, W., XU, F., LIU, C., LIU, N., CAO, M., CUI, B. & NING, G. 2013. Genetic association between IL-17F gene polymorphisms and the pathogenesis of Graves' Disease in the Han Chinese population. *Gene,* 512**,** 300-4.

JIA, H., TAO, F., LIU, C., GUO, T., ZHU, W., WANG, S., CUI, B. & NING, G. 2015. Both interleukin-23A polymorphism and serum interlukin-23 expression are associated with Graves' disease risk. *Cell Immunol,* 294**,** 39-43.

KHONG, J. J., BURDON, K. P., LU, Y., LAURIE, K., LEONARDOS, L., BAIRD, P. N., SAHEBJADA, S., WALSH, J. P., GAJDATSY, A., EBELING, P. R., HAMBLIN, P. S., WONG, R., FOREHAN, S. P., FOURLANOS, S., ROBERTS, A. P., DOOGUE, M., SELVA, D., MONTGOMERY, G. W., MACGREGOR, S. & CRAIG, J. E. 2016. Pooled genome wide association detects association upstream of FCRL3 with Graves' disease. *BMC Genomics,* 17**,** 939.

KISIEL, B., BEDNARCZUK, T., KOSTRZEWA, G., KOSINSKA, J., MISKIEWICZ, P., PLAZINSKA, M. T., BAR-ANDZIAK, E., KROLICKI, L., KRAJEWSKI, P. & PLOSKI, R. 2008. Polymorphism of the oestrogen receptor beta gene (ESR2) is associated with susceptibility to Graves' disease. *Clin Endocrinol (Oxf),* 68**,** 429-34.

KURYLOWICZ, A., KULA, D., PLOSKI, R., SKORKA, A., JURECKA-LUBIENIECKA, B., ZEBRACKA, J., STEINHOF-RADWANSKA, K., HASSE-LAZAR, K., HIROMATSU, Y., JARZAB, B. & BEDNARCZUK, T. 2005. Association of CD40 gene polymorphism (C-1T) with susceptibility and phenotype of Graves' disease. *Thyroid,* 15**,** 1119-24.

LI, H. N., LI, X. R., DU, Y. Y., YANG, Z. F. & LV, Z. T. 2020. The Association Between Foxp3 Polymorphisms and Risk of Graves' Disease: A Systematic Review and Meta-Analysis of Observational Studies. *Front Endocrinol (Lausanne),* 11**,** 392.

LI, N., ZHOU, Z., LIU, X., LIU, Y., ZHANG, J., DU, L., WEI, M. & CHEN, X. 2008. Association of tumour necrosis factor alpha (TNF-alpha) polymorphisms with Graves' disease: A meta-analysis. *Clin Biochem,* 41**,** 881-6.

LIU, L., WU, H. Q., WANG, Q., ZHU, Y. F., ZHANG, W., GUAN, L. J. & ZHANG, J. A. 2012. Association between thyroid stimulating hormone receptor gene intron polymorphisms and autoimmune thyroid disease in a Chinese Han population. *Endocr J,* 59**,** 717-23.

MITCHELL, A. L., CORDELL, H. J., SOEMEDI, R., OWEN, K., SKINNINGSRUD, B., WOLFF, A. B., ERICKSEN, M., UNDLIEN, D., HUSEBYE, E. & PEARCE, S. H. 2009. Programmed death ligand 1 (PD-L1) gene variants contribute to autoimmune Addison's disease and Graves' disease susceptibility. *J Clin Endocrinol Metab,* 94**,** 5139-45.

VELAGA, M. R., WILSON, V., JENNINGS, C. E., OWEN, C. J., HERINGTON, S., DONALDSON, P. T., BALL, S. G., JAMES, R. A., QUINTON, R., PERROS, P. & PEARCE, S. H. 2004. The codon 620 tryptophan allele of the lymphoid tyrosine phosphatase (LYP) gene is a major determinant of Graves' disease. *J Clin Endocrinol Metab,* 89**,** 5862-5.

VENETI, S., ANAGNOSTIS, P., ADAMIDOU, F., ARTZOUCHALTZI, A. M., BOBORIDIS, K. & KITA, M. 2019. Association between vitamin D receptor gene polymorphisms and Graves' disease: a systematic review and meta-analysis. *Endocrine,* 65**,** 244-251.

WAWRUSIEWICZ-KURYLONEK, N., KOPER-LENKIEWICZ, O. M., GOSCIK, J., MYSLIWIEC, J., PAWLOWSKI, P. & KRETOWSKI, A. J. 2019. Association of PTPN22 polymorphism and its correlation with Graves' disease susceptibility in Polish adult population-A preliminary study. *Mol Genet Genomic Med,* 7**,** e661.

XIONG, H., WU, M., YI, H., WANG, X., WANG, Q., NADIRSHINA, S., ZHOU, X. & LIU, X. 2016. Genetic associations of the thyroid stimulating hormone receptor gene with Graves diseases and Graves ophthalmopathy: A meta-analysis. *Sci Rep,* 6**,** 30356.

ZHANG, Q. Y., LIU, W., LI, L., DU, W. H., ZUO, C. L., YE, X. P., ZHOU, Z., YUAN, F. F., MA, Y. R., SUN, F., YU, S. S., XIE, H. J., ZHANG, C. R., YING, Y. X., YUAN, G. Y., GAO, G. Q., LIANG, J., ZHAO, S. X. & SONG, H. D. 2020. Genetic Study in a Large Cohort Supported Different Pathogenesis of Graves' Disease and Hashimoto's Hypothyroidism. *J Clin Endocrinol Metab,* 105.

ZHAO, S. X., XUE, L. Q., LIU, W., GU, Z. H., PAN, C. M., YANG, S. Y., ZHAN, M., WANG, H. N., LIANG, J., GAO, G. Q., ZHANG, X. M., YUAN, G. Y., LI, C. G., DU, W. H., LIU, B. L., LIU, L. B., CHEN, G., SU, Q., PENG, Y. D., ZHAO, J. J., NING, G., HUANG, W., LIANG, L., QI, L., CHEN, S. J., CHEN, Z., CHEN, J. L., SONG, H. D. & CHINA CONSORTIUM FOR THE GENETICS OF AUTOIMMUNE THYROID, D. 2013. Robust evidence for five new Graves' disease risk loci from a staged genome-wide association analysis. *Hum Mol Genet,* 22**,** 3347-62.
