# Supplementary material for: Cellular and molecular basis of thyroid autoimmunity
Source: Eur Thyroid J. 2021 Nov 1;11(1):e210024. doi: 10.1530/ETJ-21-0024 (PMC9142813; doi:10.1530/ETJ-21-0024)
Supplement: Supplementary Table S2. Disturbances of non-coding RNAs (ncRNA) expression and functions in autoimmune thyroid disease (AITD). AUC: area under the curve, FFPE: formalin-fixed paraffin-embedded, GD: Graves’ disease, GO: Graves’ ophthalmopathy, HHV: human herpesvirus, HT: Hashimoto’s thyroiditis, lncR [file supplementary_table_2.pdf]

Supplementary Table S2. Disturbances of non-coding RNAs (ncRNA) expression and functions in autoimmune thyroid disease (AITD). AUC: area under the curve, FFPE: formalin-fixed paraffin-embedded, GD: Graves' disease, GO: Graves' ophthalmopathy, HHV: human herpesvirus, HT: Hashimoto's thyroiditis, lncRNAs: long non-coding RNAs, PBMCs: peripheral blood mononuclear cells, TC: thyroid cancer, TgAb: thyroglobulin autoantibodies, Th: T helper cells, TPOAb: TPO autoantibodies, Treg: T regulatory lymphocyte, TRAb: TSHR autoantibodies, TSLP: thymic stromal lymphopoietin, PTC: papillary thyroid cancer.

	Expression	Samples	Functions/Association with clinical parameters	Target genes, signalling pathways	Disease	References
**microRNAs**						
miR-16	Downregulated in Treg cells while upregulated in serum of GD patients, and in T cells infected with HHV.	Treg cells of GD patients and healthy controls; serum from HT patients, GD patients and healthy controls; human thyroid and T cell lines infected *in vitro* with HHV-6A, -6B and -7.		SIN3A; NF-κβ pathway.	GD, HT	(Wang et al., 2014, Yamada et al., 2014, Caselli et al., 2017)
miR-21-5p	Upregulated in AITD thyroid and serum samples.	Fresh-frozen thyroid tissues and serum samples from AITD patients and healthy controls.	The combination of miR-21-5p and TRAb expression distinguished GD patients from healthy controls (AUC: 0.92, sensitivity: 91.7%, specificity: 87.5%). The expression of miR-21-5p positively correlated with the level of TPOAb, TgAb and TRAb. Higher expression of miR-21-5p was associated with worse GD patients' prognosis.	miR-21-5p by inhibition of Smad7 regulated the Th1/Th2 balance and stimulates Th17 differentiation.	HT, GD, GO	(Martinez-Hernandez et al., 2018, Martinez-Hernandez et al., 2019)
miR-22	Upregulated in thyroid tissue of GD patients as well as in serum of GD and GO patients.	Thyroid specimens from GD patients and healthy controls; serum samples from HT, GO and healthy controls.		EDA; GATM; MLLT4.	GD, GO, HT	(Qin et al., 2015, Yamada et al., 2014)
miR-125a	Downregulated in serum of GD patients;	Blood samples from GD (intractable, in	rs12976445 C/T polymorphism occurred	RANTES; IL-6; TGF-β; IL-23R; JAK-STAT signalling	GD, HT, thyroiditis	(Inoue et al., 2014, Peng et

	downregulated/upregulated (depending on the publication) in PBMCs of HT patients; upregulated in serum from thyroiditis mice.	remission, and uncategorized) patients, HT (severe HT, mild HT and uncategorized) patients, as well as healthy controls; PBMCs from GD patients, HT patients and healthy controls; serum from thyroiditis mice and control animals.	more frequently in patients with HT than healthy controls and in intractable GD patients compared with GD patients and GD patients in remission. Downregulation of this miRNA possibly indirectly facilitates the differentiation of Th17 cells, leading to HT development and GD intractability. The expression of miR-125a-3p inversely correlated with the level of TgAb. The expression of miR-125a-5p correlated positively with the percentage of circulating Th1 cells and the level of TPOAb. Disease biomarker distinguishes between HT patients and healthy controls (AUC: 0.74, sensitivity: 72.9%, specificity: 68%).	pathway; MAF. Inhibition of miR-125a-5p expression reduced the proportion of Th1 cells and the expression of IFN-γ in CD4+ T cells. In thyroiditis mice overexpression of miR-125a reduced autophagy of mouse macrophage cells, increased the apoptotic rate and the expression of TNF-α, IL-1β, IL-6 and IL-18.	mice	al., 2015b, Caselli et al., 2017)
miR-142-3p	Upregulated in serum from untreated GD patients compared with patients in remission and healthy controls, in AITD thyroid and serum samples, and in thyroid tissue samples from HT patients (primary HT or accompanying	Serum from GD patients (untreated and in remission) and healthy controls; thyroid tissue samples from AITD (HT, GD without GO, GD with GO) patients and	The level of miR-142-3p correlated positively with TPOAb and TgAb serum level in AITD patients.	CLDN1; Increased expression of miR-142-3p inhibited the negative regulation of CD4+C25+ T cells proliferation by Tregs.	HT, GD, GO, PTC	(Chen et al., 2018, Martinez-Hernandez et al., 2018, Zhu et al., 2016)

	PTC or nodular goitre).	healthy controls; FFPE thyroid tissues from HT patients (primary HT, HT concomitant PTC, HT concomitant nodular goitre), PTC patients, patients with nodular goitre and healthy controls.				
miR-146a	Downregulated/upregulated (depending on the publication) in serum/plasma of AITD patients and intractable GD patients as well as Treg cells of GD. Upregulated in: PBMCs of HT patients, GD patients and patients with mild HT compared with controls, thyroid samples of GD patients as well as of HT patients (primary HT or accompanying PTC or nodular goitre), and in microvesicles isolated from the blood of GD and HT patients.	Serum/plasma, Treg cells from GD patients and healthy controls; plasma and PBMCs from GD patients (intractable GD, GD in remission, and uncategorized GD), HT (severe HT, mild HT, uncategorized HT), and healthy controls; thyroid gland fine-needle aspiration biopsies from GD patients, HT patients, and healthy controls; fresh-frozen thyroid tissues from AITD patients (HT, GD without GO, GD with GO) and healthy controls.	The expression of miR-146b-3p positively correlated with the level of TRAb. Disease biomarkers (together with miR-210 and miR-155) distinguish between GD patients and healthy controls (AUC: 0.98, sensitivity: 91.3%, specificity: 93.8% with 92.4% diagnostic efficiency).	Microvesicles isolated from the blood of GD and HT patients inhibited Treg cells differentiation and the induction of Th17 cells probably through regulation of IL-8 expression via miR-146a.	GD, HT, GO, PTC	(Zheng et al., 2018, Wang et al., 2014, Otsu et al., 2017, Bernecker et al., 2014, Bernecker et al., 2012, Rodriguez-Munoz et al., 2015, Martinez-Hernandez et al., 2018, Zhu et al., 2016)
miR-146b	Upregulated in AITD fresh-frozen thyroid samples, PBMCs isolated from HT patients, thyroid tissues of patients with TC and HT compared with TC	Fresh-frozen thyroid tissues, serum samples and PBMCs from AITD patients (HT, GD without GO,		The expression of these miRNAs negatively correlated with the expression of *ENO4*, *INTU*, *KIF27*, *PACR6*, and *ITK36*	HT, GD, GO, TC	(Martinez-Hernandez et al., 2018, Martinez-Hernandez et

	or HT separately.	GD with GO), and healthy controls; tissue samples of paraneoplastic and thyroid cancers from TC patients, TC with HT patients and HT patients.		genes, associated with cilia organization.		al., 2019, Li et al., 2019, Liu et al., 2020b)
miR-155	Downregulated in: serum of GD patients, CD8+ T cells from HT patients, plasma of GD patients compared with HT, thyroid samples of HT patients, and in human thyroid cells infected with HHV.<br>Upregulated in: plasma of patients with severe HT, Treg cells of GD patients, microvesicles isolated from blood of GD and HT patients, as well as in AITD thyroid samples.	Serum and Treg cells from GD patients and healthy controls; plasma and PBMCs from GD patients (intractable, in remission, and uncategorized), HT patients (severe, mild, uncategorized) and healthy controls; thyroid gland fine-needle aspiration biopsies from GD, HT patients, and healthy controls; CD4+ and CD8+ T cells from HT and GD patients as well as healthy controls; fresh-frozen thyroid tissues and serum samples from AITD patients (HT, GD without GO, GD with GO) and healthy controls; human thyroid and T cell lines infected *in vitro* with HHV-6A, -6B and -7.	Disturbed expression of miR-155 in serum of GD patients was linked with the extent of goitre; disease biomarkers (together with miR-210 and miR-146a) distinguish between GD patients and healthy controls (AUC: 0.98, sensitivity: 91.3%, specificity: 93.8% with 92.4% diagnostic efficiency); HHV-6A infection leads to disturbances in the expression of AITD-associated miRNAs and, in consequence might stimulate AITD development.	Downregulation of this miRNA in CD8+ T cells might lead to pathological identification of thyroid-specific antigens by these cells. Microvesicles isolated from the blood of GD and HT patients inhibited Treg cells differentiation and the induction of Th17 cells probably through regulation of SMAD4 expression via miR-155.	GD, HT, GO	(Zheng et al., 2018, Wang et al., 2014, Otsu et al., 2017, Bernecker et al., 2014, Bernecker et al., 2012, Rodriguez-Munoz et al., 2015, Martinez-Hernandez et al., 2018, Caselli et al., 2017)

miR-200a	Upregulated in thyroid samples of HT patients; downregulated in CD4+ and CD8+ T cells from HT and GD patients.	Thyroid gland fine-needle aspiration biopsies, PBMCs as well as CD4+ and CD8+ T cells from GD, HT patients and healthy controls.		Downregulation of these miRNAs in CD8+ T cells of HT patients possibly results in a more significant production of proinflammatory Th1 cytokines and destroy thyroid cells. In contrast, a decrease of their expression in CD8+ T cells might contribute to improper recognition of thyroid-specific antigens and AITDs development.	GD, HT	(Bernecker et al., 2014, Bernecker et al., 2012)
miR-326	Upregulated in PBMCs from HT patients as well as in thyroid tissue samples from mice with autoimmune thyroiditis.	PBMCs from HT patients and healthy controls; thyroid samples and Th17 cells from mice with autoimmune thyroiditis and healthy animals.	The expression of miR-326 positively correlated with the serum level of TgAb and TPOAb of HT patients.	miR-326 by regulation of ADAM17 expression affected IL-23/IL-23R/Th17 pathway, which leads to stimulation of Th17 cells differentiation. miR-326 by inhibition of ETS-1 expression increases the level of Th17 cells.	HT, thyroiditis mice	(Liu et al., 2020a, Zhao et al., 2018)
miR-375	Upregulated in serum and plasma of GD and HT patients.	Serum from HT patients, GD patients and healthy controls; plasma samples from HT patients and healthy controls.	Disease biomarker (together with miR-205, miR-20a-3p, miR-296, miR-451, and miR-500a) distinguish between HT patients and healthy controls (AUC: 0.75, sensitivity: 0.75, specificity: 0.66). The expression of miR-375a correlated with lower TSH level.	Regulates TSLP expression, playing key role in the stimulation of $CD4^+$ T cells differentiation into Th2 and Th17 cells.	GD, HT	(Yamada et al., 2014, Zhao et al., 2018)
miR-431*	Downregulated in PBMCs from	PBMCs from GD		Treatment of PBMCs	GD	(Liu et al.,

	initial (untreated) GD patients compared with GD in remission and healthy controls. Upregulated in T cells from PBMCs of GD.	(initial and in remission) patients and healthy controls; CD4+ and CXCR5+ T cells from PBMCs of GD patients and healthy controls.		delivered from healthy subjects with T3 leads to downregulation of these miRNA.		2012, Chen et al., 2015)
miR-451	Upregulated in serum and plasma of GD and HT patients, and in T cells infected with HHV.	Serum from HT, GD patients and healthy controls; plasma samples from HT patients and healthy controls; human thyroid and T cell lines infected *in vitro* with HHV-6A, -6B and -7.	Disease biomarkers (together with miR-205, miR-20a-3p, miR-296, miR-500a) distinguish between HT patients and healthy controls (AUC: 0.75, sensitivity: 0.75, specificity: 0.66). The expression of miR-451 correlated with higher TSH level.	Cell death pathway	GD, HT	(Yamada et al., 2014, Zhao et al., 2018, Caselli et al., 2017)
**lncRNAs**						
n335641; TCONS-00022357-XLOC-010919	Upregulated in PBMCs from blood of GD patients.	PBMCs from GD patients and healthy controls.	These lncRNAs via regulation of TCL1A and SH2D1A expression might regulate B cells proliferation and survival and thus participate in GD development.	TCL1A	GD	(Jiang et al., 2020)
n337845	Downregulated in PBMCs from blood of GD patients.			SH2D1A		
AB075506; AL832122; AK055670; AF318328; AK021954	Upregulated in the CD4+ T cells of initial GD patients.	PBMCs from GD patients (untreated, euthyroid as well as in remission) and healthy controls.	The expression of AK021954 and AB075506 correlated positively while HMlincRNA1474 negatively with serum level of FT3, FT4 and TRAb. Additionally, the expression of AK021954	The expression of HMlincRNA1474 correlated positively with JUNB expression while the level of AK021954 and AB075506 with NRCAM expression.	GD	(Yin et al., 2020)
HMlincRNA1474; TCONS-00012608; AK126108	Downregulated in the CD4+ T cells of initial GD patients.					

			and AB075506 correlated negatively with TSH level. Moreover, the level of TCONS-00012608 was negatively associated with the level of FT3, FT4 and TRAb. Diagnostic biomarkers distinguish between GD patients and healthy controls: AUC for AK021954: 0.81, for AB075506: 0.76, and for HMlincRNA1474: 0.81.			
IFNG-AS1	Upregulated in PBMCs and thyroid tissue samples from HT patients.	Blood and thyroid tissue samples from HT patients and healthy controls.	The expression of IFNG-AS1 correlated positively with the percentage of Th1 cells and the level of TgAb as well as TPOAb.	Downregulation of IFNG-AS1 expression resulted in a decline of percentage of IFN-γ+ cells.	HT	(Peng et al., 2015a)
XLOC_I2_006631; LOC729737	Upregulated in PBMCs from HT patients.	PBMCs from HT patients and healthy controls.	The expression of XLOC_I2_006631 correlated positively with TPOAb level. Disease biomarkers distinguish between HT patients and healthy controls. (XLOC_I2_006631: AUC: 0.85, sensitivity: 88.9%, specificity: 75%; LOC729737: AUC: 0.83, sensitivity: 74.1%, specificity: 89.3%).	NF-κβ; TGF-β signalling pathway; MECP2. The expression of MECP2 increased in HT patients and correlated positively with the expression of XLOC_I2_006631 and the level of TPOAb.	HT	(Peng et al., 2020)
BC041964	Downregulated in PBMCs from					

	HT patients.					
			**circRNAs**			
circ_0089172; circ_0007777; circ_0012152; circ_0000075	Upregulated in PBMCs of HT patients.	Blood samples from HT patients and healthy controls.	The expression of circ_0089172 correlated positively with the serum level of TPOAb. Disease biomarkers distinguish between HT patients and healthy controls (AUC for circ_0089172: 0.67, circ_0012152: 0.70, and circ_0000075: 0.72).	IL-23R; circ_0089172 play the role of miR-125a-3p sponge. Downregulation of circ_0089172 expression leads to overexpression of miR-125a-3p and decline in IL-23R expression.	HT	(Xiong et al., 2019)

References:

BERNECKER, C., HALIM, F., LENZ, L., HAASE, M., NGUYEN, T., EHLERS, M., VORDENBAEUMEN, S. & SCHOTT, M. 2014. microRNA expressions in CD4+ and CD8+ T-cell subsets in autoimmune thyroid diseases. Exp Clin Endocrinol Diabetes, 122, 107-12.

BERNECKER, C., LENZ, L., OSTAPCZUK, M. S., SCHINNER, S., WILLENBERG, H., EHLERS, M., VORDENBAUMEN, S., FELDKAMP, J. & SCHOTT, M. 2012. MicroRNAs miR-146a1, miR-155_2, and miR-200a1 are regulated in autoimmune thyroid diseases. Thyroid, 22, 1294-5.

CASELLI, E., D'ACCOLTI, M., SOFFRITTI, I., ZATELLI, M. C., ROSSI, R., DEGLI UBERTI, E. & DI LUCA, D. 2017. HHV-6A in vitro infection of thyrocytes and T cells alters the expression of miRNA associated to autoimmune thyroiditis. Virol J, 14, 3.

CHEN, J., TIAN, J., TANG, X., RUI, K., MA, J., MAO, C., LIU, Y., LU, L., XU, H. & WANG, S. 2015. MiR-346 regulates CD4(+)CXCR5(+) T cells in the pathogenesis of Graves' disease. Endocrine, 49, 752-60.

CHEN, X., HUANG, F., QI, Y., ZHOU, M., YIN, Q., PENG, Y., ZHOU, Y., NING, G. & WANG, S. 2018. Serum and thyroid tissue level of let-7b and their correlation with TRAb in Graves' disease. J Transl Med, 16, 188.

INOUE, Y., WATANABE, M., INOUE, N., KAGAWA, T., SHIBUTANI, S., OTSU, H., SAEKI, M., TAKUSE, Y., HIDAKA, Y. & IWATANI, Y. 2014. Associations of single nucleotide polymorphisms in precursor-microRNA (miR)-125a and the expression of mature miR-125a with the development and prognosis of autoimmune thyroid diseases. Clin Exp Immunol, 178, 229-35.

JIANG, X., WANG, Y., LI, X., HE, L., YANG, Q., WANG, W., LIU, J. & ZHA, B. 2020. Microarray profile of B cells from Graves' disease patients reveals biomarkers of proliferation. Endocr Connect, 9, 405-417.

LI, N., LIU, X., HAN, L., ZHOU, R., YAN, J., ZHAO, G. & LIU, L. 2019. Expression of miRNA-146b-5p in patients with thyroid cancer in combination with Hashimoto's disease and its clinical significance. Oncol Lett, 17, 4871-4876.

LIU, R., MA, X., XU, L., WANG, D., JIANG, X., ZHU, W., CUI, B., NING, G., LIN, D. & WANG, S. 2012. Differential microRNA expression in peripheral blood mononuclear cells from Graves' disease patients. J Clin Endocrinol Metab, 97, E968-72.

LIU, Y., CUI, X., WANG, S., LIU, J., ZHAO, N., HUANG, M., QIN, J., LI, Y., SHAN, Z. & TENG, W. 2020a. Elevated MicroRNA-326 Levels Regulate the IL-23/IL-23R/Th17 Cell Axis in Hashimoto's Thyroiditis by Targeting a Disintegrin and Metalloprotease 17. Thyroid, 30, 1327-1337.

LIU, Y., DING, X., XIONG, S., WANG, X., TANG, X., WANG, L., WANG, S. & PENG, H. 2020b. Circulating microRNA Expression Profiling Identifies miR-125a-5p Promoting T Helper 1 Cells Response in the Pathogenesis of Hashimoto's Thyroiditis. Front Immunol, 11, 1195.

MARTINEZ-HERNANDEZ, R., SAMPEDRO-NUNEZ, M., SERRANO-SOMAVILLA, A., RAMOS-LEVI, A. M., DE LA FUENTE, H., TRIVINO, J. C., SANZ-GARCIA, A., SANCHEZ-MADRID, F. & MARAZUELA, M. 2018. A MicroRNA Signature for Evaluation of Risk and Severity of Autoimmune Thyroid Diseases. J Clin Endocrinol Metab, 103, 1139-1150.

MARTINEZ-HERNANDEZ, R., SERRANO-SOMAVILLA, A., RAMOS-LEVI, A., SAMPEDRO-NUNEZ, M., LENS-PARDO, A., MUNOZ DE NOVA, J. L., TRIVINO, J. C., GONZALEZ, M. U., TORNE, L., CASARES-ARIAS, J., MARTIN-COFRECES, N. B., SANCHEZ-MADRID, F. & MARAZUELA, M. 2019. Integrated miRNA and mRNA expression profiling identifies novel targets and pathological mechanisms in autoimmune thyroid diseases. EBioMedicine, 50, 329-342.

OTSU, H., WATANABE, M., INOUE, N., MASUTANI, R. & IWATANI, Y. 2017. Intraindividual variation of microRNA expression levels in plasma and peripheral blood mononuclear cells and the associations of these levels with the pathogenesis of autoimmune thyroid diseases. Clin Chem Lab Med, 55, 626-635.

PENG, H., LIU, Y., TIAN, J., MA, J., TANG, X., RUI, K., TIAN, X., MAO, C., LU, L., XU, H., JIANG, P. & WANG, S. 2015a. The Long Noncoding RNA IFNG-AS1 Promotes T Helper Type 1 Cells Response in Patients with Hashimoto's Thyroiditis. Sci Rep, 5, 17702.

PENG, H., LIU, Y., TIAN, J., MA, J., TANG, X., YANG, J., RUI, K., ZHANG, Y., MAO, C., LU, L., XU, H. & WANG, S. 2015b. Decreased expression of microRNA-125a-3p upregulates interleukin-23 receptor in patients with Hashimoto's thyroiditis. Immunol Res, 62, 129-36.

PENG, H., XIONG, S., DING, X., TANG, X., WANG, X., WANG, L. & LIU, Y. 2020. Long noncoding RNA expression profiles identify lncRNAXLOC_I2_006631 as a potential novel blood biomarker for Hashimoto's thyroiditis. Int J Mol Med, 46, 2172-2184.

QIN, Q., WANG, X., YAN, N., SONG, R. H., CAI, T. T., ZHANG, W., GUAN, L. J., MUHALI, F. S. & ZHANG, J. A. 2015. Aberrant expression of miRNA and mRNAs in lesioned tissues of Graves' disease. Cell Physiol Biochem, 35, 1934-42.

RODRIGUEZ-MUNOZ, A., MARTINEZ-HERNANDEZ, R., RAMOS-LEVI, A. M., SERRANO-SOMAVILLA, A., GONZALEZ-AMARO, R., SANCHEZ-MADRID, F., DE LA FUENTE, H. & MARAZUELA, M. 2015. Circulating Microvesicles Regulate Treg and Th17 Differentiation in Human Autoimmune Thyroid Disorders. J Clin Endocrinol Metab, 100, E1531-9.

WANG, Z., FAN, X., ZHANG, R., LIN, Z., LU, T., BAI, X., LI, W., ZHAO, J. & ZHANG, Q. 2014. Integrative analysis of mRNA and miRNA array data reveals the suppression of retinoic acid pathway in regulatory T cells of Graves' disease. J Clin Endocrinol Metab, 99, E2620-7.

XIONG, S., PENG, H., DING, X., WANG, X., WANG, L., WU, C., WANG, S., XU, H. & LIU, Y. 2019. Circular RNA Expression Profiling and the Potential Role of hsa_circ_0089172 in Hashimoto's Thyroiditis via Sponging miR125a-3p. Mol Ther Nucleic Acids, 17, 38-48.

YAMADA, H., ITOH, M., HIRATSUKA, I. & HASHIMOTO, S. 2014. Circulating microRNAs in autoimmune thyroid diseases. Clin Endocrinol (Oxf), 81, 276-81.

YIN, L., ZENG, C., YAO, J. & SHEN, J. 2020. Emerging Roles for Noncoding RNAs in Autoimmune Thyroid Disease. Endocrinology, 161.

ZHAO, N., ZOU, H., QIN, J., FAN, C., LIU, Y., WANG, S., SHAN, Z., TENG, W. & LI, Y. 2018. MicroRNA-326 contributes to autoimmune thyroiditis by targeting the Ets-1 protein. Endocrine, 59, 120-129.

ZHENG, L., ZHUANG, C., WANG, X. & MING, L. 2018. Serum miR-146a, miR-155, and miR-210 as potential markers of Graves' disease. J Clin Lab Anal, 32.

ZHU, J., ZHANG, Y., ZHANG, W., ZHANG, W., FAN, L., WANG, L., LIU, Y., LIU, S., GUO, Y., WANG, Y., YI, J., YAN, Q., WANG, Z. & HUANG, G. 2016. MicroRNA-142-5p contributes to Hashimoto's thyroiditis by targeting CLDN1. J Transl Med, 14, 166.
