# Supplementary material for: Cellular and molecular basis of thyroid autoimmunity
Source: Eur Thyroid J. 2021 Nov 1;11(1):e210024. doi: 10.1530/ETJ-21-0024 (PMC9142813; doi:10.1530/ETJ-21-0024)
Supplement: Supplementary Table S3. The changes in composition of gut microbiota in AITD patients. Regarding GD, most studies report higher proportion of Bacteroidetes and lower of Firmicutes (at the phyla level) whereas higher number of Bacteroides, Lactobacillus and Prevotella (at the genus level) (Ishaq et a [file supplementary_table_3.pdf]

Supplementary Table S3. The changes in composition of gut microbiota in AITD patients. Regarding GD, most studies report higher proportion of Bacteroidetes and lower of Firmicutes (at the phyla level) whereas higher number of *Bacteroides*, Lactobacillus and *Prevotella* (at the genus level) (Ishaq et al., 2018, Jiang et al., 2021). The abundance of genera such as *Blautia*, *Eubacterium helii*_group, *Lactobacillus* and *Dorea* distinguished GD patients from healthy controls, suggestive of the potential diagnostic significance. The number of *Blautia* correlated positively, while *Dorea* correlated negatively with serum level of TPOAb, indicating clinical significance of these findings (Jiang et al., 2021). Similarly, the abundance of Proteobacteria, Tenericutes and Synergistetes correlated negatively with FT3, FT4, TPOAb and TRAb levels in GD patients (Su et al., 2020). These changes may have biological significance, since Ishaq et al. showed that supernatant of medium used for culturing of *B. fragilis* (of which proportion is reduced in GD patients) increased the percentage of $CD4^{+}$ $CD25^{+}$ $FOXP3^{+}$ Treg cells and the level of IL-10, while suppressed the percentage of $CD4^{+}$ $IL17^{+}$ Th17 cells and the level of IL-17A in PBMCs from healthy controls (Su et al., 2020). The reciprocal links between thyroid function and microbiome are further supported by studies GD patients treated with antithyroid drugs, PTU or methimazole (MMI), which significantly changed the structure of gut microbiota (Sun et al., 2020). In HT patients, key microbiota alterations include increased proportion of gut Firmicutes and reduced number of Bacteroidetes. At the genus level, the abundance of *Blautia*, *Roseburia*, *Romboutia* and *Dorea* was increased, whereas the number of *Faecalibacterium* and *Prevotella* was lower in HT patients when compared with controls (Zhao et al., 2018). The composition of the intestinal microflora also correlates with the clinical parameters of HT patients and response to LT4 treatments. Specifically, the number of *Lechnospiraceae*, *Fusicatenibacter* and additional 13 genera were associated with TPOAb level (Zhao et al., 2018), while the proportion of *Clostridium coccoides* correlated with TSH level and the time of HT disease duration. The abundance of *Bacteroides*, *Faecalibacterium*, *Prevotella* and additional 7 genera was suggested as a promising biomarker distinguishing HT patients from healthy controls (Zhao et al., 2018).

**Composition of gut microbiota.**	**Samples; population.**	**Association between microbiota and clinical data.**	**Disease**	**Reference**
Bacterial richness: (SOBs, ACE and Chao1 indexes) reduced in GD patients.<br>Bacterial α diversity: Shannon index reduced in GD.<br>Bacterial β diversity: (PLS-DA; Bray-Curtis index): Significant differences between microbiome of GD and healthy controls.<br>Phyla level: Nitrospinae and Spirochaetes are unique for GD patients. Higher abundance of Bacteroidetes and Saccharibacteria while lower of Firmicutes, Synergistetes, Proteobacteria, Verrucomicrobiota, and Tenericutes.<br>Genus level: *Negativicoccus, Bacillus*, *Succiniclastricum, Campylobacterales* and	Faecal and blood samples from GD (GD with HT, GD without HT) untreated patients as well as from GD patients (HG – group before treatment; TG-group after treatment with methimazole for 3-5 months) and healthy controls.<br>Chinese population.	Analysis of abundance of Bacteroides, *Blautia*, *Eubacterium hellii*_group, *Anaerostipes*, *Lactobacillus*, *Dorea, Peptostreptococcaceae*, *Collinsella* and *Ruminococcus_torques*_group might serve as diagnostic biomarkers distinguish GD patients from healthy controls (AUC: 0.8109).<br>The number of *Blautia* correlated positively while *Bacteroides* negatively with the level of TPOAb and TMAb. Moreover, *Dorea* negatively correlated with the level of TPOAb.<br>The number of Synergistetes correlated negatively with the level of TRAb, TGAb and TPOAb, while the number of *Lactobacillus* positively with the level of TRAb and TPOAb. The level of TRAb correlated negatively also with the number of *Phascolarctobacterium*. After	GD	(Jiang et al., 2021, Ishaq et al., 2018, Chen et al., 2021, Su et al., 2020, Yan et al., 2020)

Gastranaerophilales are unique for GD patients. Higher proportion of *Bacteroides, Lactobacillus, Prevotella, Veillonella* and *Phascolarctobacterium,* while lower of *Blautia, Anaerostipes, Collinsella, Dorea, Alistipes, Ryminococcus,* and *Faecalibacterium* in GD.<br>Higher proportion of *Blautia*, *Streptococcus*, and *Ruminococcus*, while lower of *Phascolarctobacterium* and *Lachnospira* in TG vs HG.		treatment of GD patients the relative abundance of *Ruminococcus* correlated positively, while *Phascolarctobacterium* negatively with the level of TRAb. The number of Proteobacteria, Tenericutes, Verrucomicrobia and Synergistetes correlated negatively with the serum levels of FT3, FT4, TPOAb and TRAb, while positively with TSH level. Contrary, Bacteroidetes and Saccharibacteria correlated positively with the level of FT3, FT4, TPOAb and TRAb, while negatively with the level of TSH.		
Bacterial richness: (ACE and Chao1 indexes) No differences.<br>Bacterial α diversity: Shannon index reduced in GD and GO patients.<br>Bacterial β diversity: (PCoA) Significant differences between GD patients and healthy controls.<br>Phyla level: Deinococcus-Thermus Chloroflexi decreased in GO compared with GD.<br>Genus level: *Subdoligranulum* and *Bilophila* augmented while *Blautia, Anaerostipes, Dorea, Butyricicoccus, Romboutsia, Fusicatenibacter, Collinesella, Intestinibacter,* and *Phascolarclobacterium* decreased in GO patients compared with GD.	Faecal samples form GD patients without GO, GO patients and healthy controls. Chinese population.		GD, GO	(Shi et al., 2019)
Bacterial richness: (ACE and Chao1 indexes) No differences.<br>Bacterial α diversity: Shannon and Simpson indexes are reduced in GO compared with healthy controls.<br>Bacterial β diversity: (PCoA and ANOSIM) Significant differences in microbiome composition between GO patients and	Faecal samples from patients with severe and active GO and healthy controls. Chinese population.	At the family level the proportion of *Succinivibrionaceae* correlated positively with TRAb; at the genus such correlation was observed between the number of *Subdoligranulum* and TRAb. The number of *Prevotellaceae* is disease biomarker which distinguish GO patients from healthy controls (AUC: 0.755).	GO	(Shi et al., 2019)

healthy controls.<br><u>Phyla level:</u> Bacteroidetes are more enriched in AITD vs healthy controls. Firmicutes, Proteobacteria, and Actinobacteria are reduced in GO patients compared with healthy controls.<br><u>Genus level:</u> Higher proportion of *Prevotellaceae* while lower of *Blautia, Fusicatenibacter, Butyricicoccus, Anaerostipes,* and *Collinsella* in GO patients compared with healthy controls.				
<u>Bacterial α diversity:</u><br>No differences or lower level (depending on publication) of Shannon index in AITD vs. healthy controls, no differences between HT and GD.<br><u>Bacterial β diversity:</u> (PCoA) Significant differences in microbiome composition between GD and HT vs healthy controls.<br><u>Genus level:</u><br>*Fusobacterium was* more enriched while *Faecalibacterium* reduced in GD compared with HT and controls. Higher abundance of *Sutterella* in GD compared with controls. Decreased number of *Rikenellaceae* in GD compared with HT.	Faecal and blood samples from GD, HT patients and healthy controls. Spain and Egyptian populations.	The number of Alistipes, Ruminococcaceae and Enterobacteriaceae correlated positively while Faecalibacterium negatively with the level of TPOAb. Moreover, the number of Lactobacillaceae, *Lactobacillus* and *Pasteurellaceae* correlated positively while *Paecalibacterium* negatively with the level of TSIAb (thyroid stimulating immunoglobulin antibody). The abundance of Bacteroidetes and Firmicutes correlated positively with the level of TRAb, while Bacteroidetes, Firmicutes and Prevotella correlated positively with TPOAb level.	GD, HT	(Cornejo-Pareja et al., 2020, El-Zawawy et al., 2021)
<u>Bacterial α diversity:</u> Reduced Shannon index in HT vs controls, and in hypothyroid group vs euthyroid.<br><u>Bacterial β diversity:</u> (PCoA, ANOSIM) Significant differences in microbiome between HT and healthy controls.<br><u>Phyla level:</u> Firmicutes augmented while Bacteroidetes reduced in HT vs controls.<br><u>Genus level:</u> Higher abundance of *Bacteroides* and *Prevotella* in hypothyroid	Faecal and blood samples from HT patients (euthyroid and hypothyroid) and healthy controls.<br>Chinese and Brazil populations.	The number of *Lechnospiraceae*, *Fusicatenibacter*, *Anaerostipes*, *Eubacterium hallii_group*, *Blantia* and additional 10 genera correlated positively with the level of TPOAb while the number of *Prevotella_9*, *Bacteroides*, *Phascolactobacterium* and *Paraprevotella* negatively with the levels of these antibodies. Moreover, the number of *Fusicatenibacter* correlated negatively while *Alloprevotella* positively with the level of FT4, and with *Romboutsia* correlated negatively with the level of TSH. The analysis of *Bacteroides,*	HT	(Liu et al., 2020, Zhao et al., 2018, Cayres et al., 2021)

group vs euthyroid and controls. More enriched *Lechnospiraceae_incertae_sedis, Lactorifactor, Alistipes,* and *Subdoligranulum* in euthyroid group vs controls. More enriched *Phascolarctobacterium* in hypothyroid group vs controls. Higher proportion of *Blautia, Roseburia, Ruminococcus_torque_group, Romboutia, Dorea, and Fusicatenibacter while lower of Faecalibacterium, Bacteroides, Prevotella_9, and Lachnoclostridium* in HT vs controls.		*Faecalibacterium, Prevotella_9, Blautia, Eubacterium_hallii_group, Ruminococcus_torques_group, Streptococcus, Alloprevotella, Roseburia* and *Fusicatenibacter* abundance distinguish HT patients from healthy controls (AUC:0.88).<br>The REUs of *Clostridium coccoides* and *Clostridium coccoides-Eubacteria rectale* correlated positively with TSH level and with the time of HT disease duration. Moreover, the REUs of *Roseburia* species correlated negatively with FT4 levels. Additionally, the REUs of *Lactobacillus* differed between patients treated with LT4 and untreated.		

References:

CAYRES, L. C. F., DE SALIS, L. V. V., RODRIGUES, G. S. P., LENGERT, A. V. H., BIONDI, A. P. C., SARGENTINI, L. D. B., BRISOTTI, J. L., GOMES, E. & DE OLIVEIRA, G. L. V. 2021. Detection of Alterations in the Gut Microbiota and Intestinal Permeability in Patients With Hashimoto Thyroiditis. *Front Immunol,* 12**,** 579140.

CHEN, J., WANG, W., GUO, Z., HUANG, S., LEI, H., ZANG, P., LU, B., SHAO, J. & GU, P. 2021. Associations between gut microbiota and thyroidal function status in Chinese patients with Graves' disease. *J Endocrinol Invest*.

CORNEJO-PAREJA, I., RUIZ-LIMON, P., GOMEZ-PEREZ, A. M., MOLINA-VEGA, M., MORENO-INDIAS, I. & TINAHONES, F. J. 2020. Differential Microbial Pattern Description in Subjects with Autoimmune-Based Thyroid Diseases: A Pilot Study. *J Pers Med,* 10.

EL-ZAWAWY, H. T., AHMED, S. M., EL-ATTAR, E. A., AHMED, A. A., ROSHDY, Y. S. & HEADER, D. A. 2021. Study of gut microbiome in Egyptian patients with autoimmune thyroid diseases. *Int J Clin Pract,* 75**,** e14038.

ISHAQ, H. M., MOHAMMAD, I. S., SHAHZAD, M., MA, C., RAZA, M. A., WU, X., GUO, H., SHI, P. & XU, J. 2018. Molecular Alteration Analysis of Human Gut Microbial Composition in Graves' disease Patients. *Int J Biol Sci,* 14**,** 1558-1570.

JIANG, W., YU, X., KOSIK, R. O., SONG, Y., QIAO, T., TONG, J., LIU, S., FAN, S., LUO, Q., CHAI, L., LV, Z. & LI, D. 2021. Gut Microbiota May Play a Significant Role in the Pathogenesis of Graves' Disease. *Thyroid,* 31**,** 810-820.

LIU, S., AN, Y., CAO, B., SUN, R., KE, J. & ZHAO, D. 2020. The Composition of Gut Microbiota in Patients Bearing Hashimoto's Thyroiditis with Euthyroidism and Hypothyroidism. *Int J Endocrinol,* 2020**,** 5036959.

SHI, T. T., XIN, Z., HUA, L., ZHAO, R. X., YANG, Y. L., WANG, H., ZHANG, S., LIU, W. & XIE, R. R. 2019. Alterations in the intestinal microbiota of patients with severe and active Graves' orbitopathy: a cross-sectional study. *J Endocrinol Invest,* 42**,** 967-978.

SU, X., YIN, X., LIU, Y., YAN, X., ZHANG, S., WANG, X., LIN, Z., ZHOU, X., GAO, J., WANG, Z. & ZHANG, Q. 2020. Gut Dysbiosis Contributes to the Imbalance of Treg and Th17 Cells in Graves' Disease Patients by Propionic Acid. *J Clin Endocrinol Metab,* 105.

SUN, J., ZHAO, F., LIN, B., FENG, J., WU, X., LIU, Y., ZHAO, L., ZHU, B. & WEI, Y. 2020. Gut Microbiota Participates in Antithyroid Drug Induced Liver Injury Through the Lipopolysaccharide Related Signaling Pathway. *Front Pharmacol,* 11**,** 598170.

YAN, H. X., AN, W. C., CHEN, F., AN, B., PAN, Y., JIN, J., XIA, X. P., CUI, Z. J., JIANG, L., ZHOU, S. J., JIN, H. X., OU, X. H., HUANG, W., HONG, T. P. & LYU, Z. H. 2020. Intestinal microbiota changes in Graves' disease: a prospective clinical study. *Biosci Rep,* 40.

ZHAO, F., FENG, J., LI, J., ZHAO, L., LIU, Y., CHEN, H., JIN, Y., ZHU, B. & WEI, Y. 2018. Alterations of the Gut Microbiota in Hashimoto's Thyroiditis Patients. *Thyroid,* 28**,** 175-186.
